# The unique architecture of umbrella toxins permits a two-tiered molecular bet hedging strategy for interbacterial antagonism

**DOI:** 10.1016/j.cell.2025.10.044

**Published:** 2025-12-02

**Authors:** Qinqin Zhao, Jiri Vlach, Young-Jun Park, Yongjun Tan, Savannah K. Bertolli, Pooja Srinivas, Pinyu Liao, Connor R. Fitzpatrick, Jeffery L. Dangl, Parastoo Azadi, Frank DiMaio, S. Brook Peterson, Dapeng Zhang, David Veesler, Joseph D. Mougous

**Affiliations:** 1 Department of Microbiology, University of Washington, Seattle, WA, USA; 2 Complex Carbohydrate Research Center, University of Georgia, Athens, Georgia, USA; 3 Department of Biochemistry, University of Washington, Seattle, WA, USA; 4 Howard Hughes Medical Institute, University of Washington, Seattle, WA, USA; 5 Department of Biology, Saint Louis University, St. Louis, MO, USA; 6 Department of Biology, University of North Carolina at Chapel Hill, Chapel Hill, NC, USA; 7 Howard Hughes Medical Institute, University of North Carolina at Chapel Hill, Chapel Hill, NC, USA; 8 Institute for Protein Design, University of Washington, Seattle, WA USA; 9 Program of Bioinformatic and Computational Biology, St. Louis University, St. Louis, MO USA; 10 Microbial Interactions and Microbiome Center, University of Washington, Seattle, WA, USA

## Abstract

Bacteria exist in competitive and rapidly changing environments in which the nature of future threats cannot be easily predicted. *Streptomyces coelicolor* produces three antibacterial umbrella particles harboring distinct polymorphic toxin domains and an overlapping set of six diversified lectins. Here we show that the exquisite specificity of umbrella particles derives from lectin-mediated species-specific binding to previously undescribed hypervariable surface glycoconjugates. A cryo-EM structure of one such lectin in complex with its oligosaccharide substrate defines the molecular basis for targeting through coordinated recognition of multiple glycan features. Biochemical and genetic studies of several target species, in conjunction with lectin swapping experiments, support a model whereby *S. coelicolor* umbrella toxin diversification at the levels of lectin composition and toxin polymorphism represents a unique, two-tiered bet hedging strategy. Bioinformatic analyses support this as a means by which the unusual architecture of umbrella toxins offers *Streptomyces* a generalizable strategy to antagonize an unpredictable array of competitors.

## Mougus in brief

*Streptomyces* bacteria employ a versatile two-tiered survival strategy by producing diverse umbrella toxins with highly specific lectins that recognize hypervariable sugars on competing microbes. Structural and genetic studies demonstrate that this adaptive mechanism allows the bacteria to effectively target and antagonize a wide range of rivals in unpredictable, competitive environments.

## Graphical Abstract

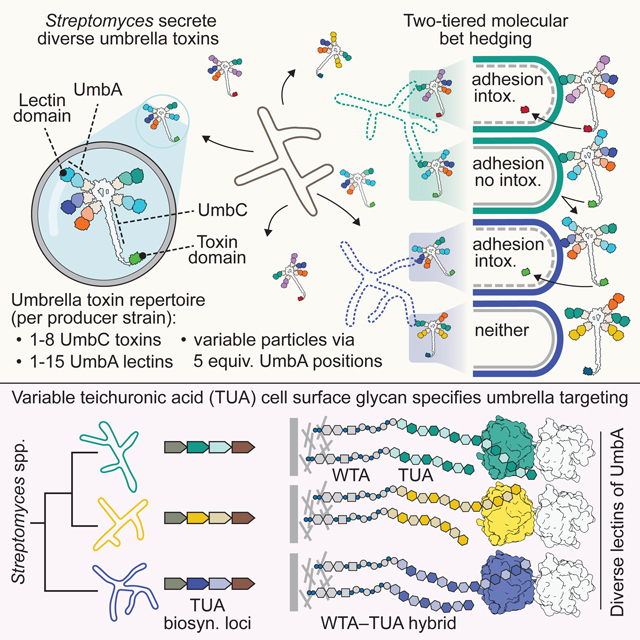

## Introduction

Microbial life is shaped by relentless competition, with bacteria vying for limited resources and access to physical niches. One manifestation of this competition is the evolution of interbacterial toxins, which exhibit remarkable diversity and act through a wide range of delivery systems and inhibitory mechanisms^1–8^. Many diffusible toxins require specific receptors on the surface of target cells—a feature that enables precise delivery but also makes them susceptible to evasion^9–11^. These constraints have fueled molecular arms races between toxin-producing bacteria and their targets^12^. The effectiveness of secreted toxins is further influenced by the inherent unpredictability of the immediate microenvironment, where the identity of competing bacteria can vary substantially^13^. This could conceivably lead to a bet hedging-type evolutionary strategy, where individual cells in the population might secrete variable assortments of toxins or the toxin molecules themselves could possess features that hedge against unpredictable variability in their targets^14,15^.

**Figure 1.**
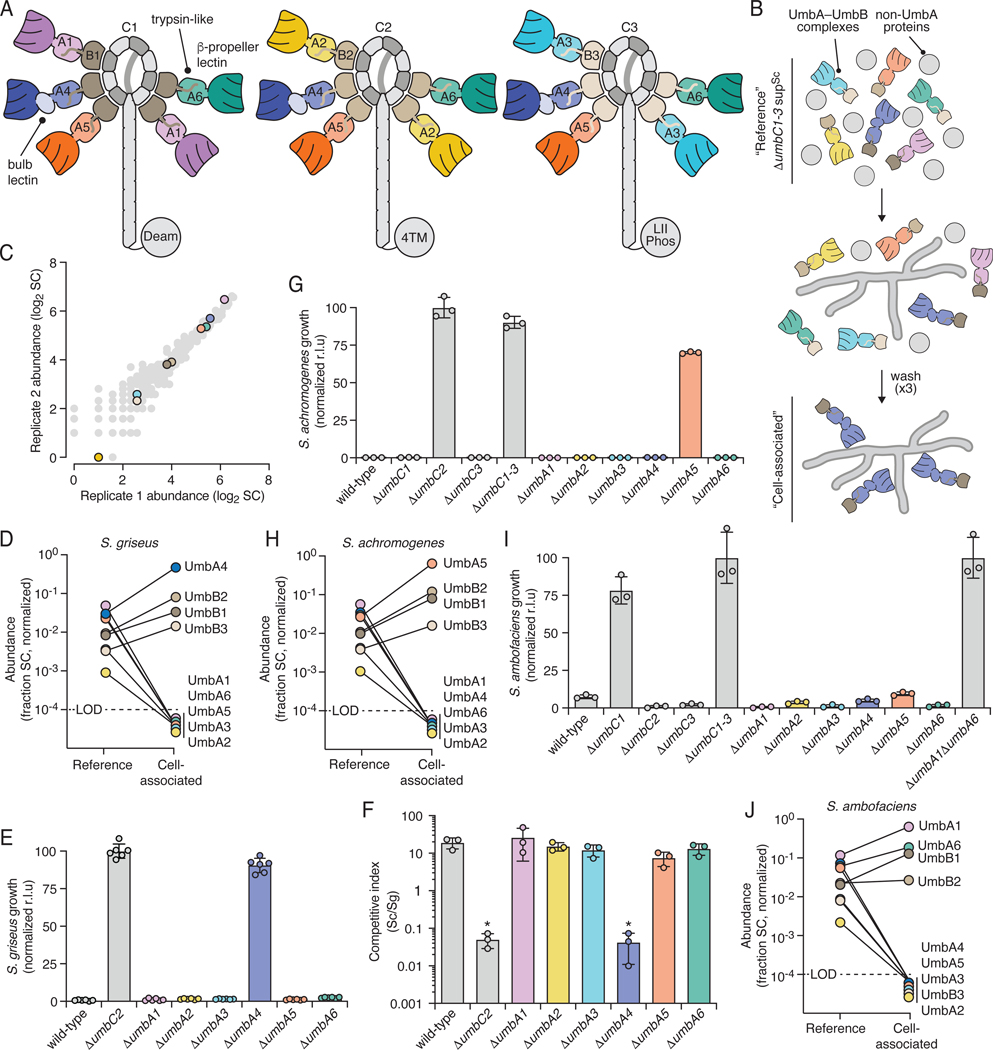
UmbA proteins produced by *S. coelicolor* selectively bind target species. A) Schematic illustrating the composition of the three umbrella toxin particles produced by *S. coelicolor.* The complement of UmbA proteins associated with a given particle can vary; schematics depict two cognate UmbA proteins on their respective particle (UmbA1–3) and one of each orphan UmbA protein that bind all particles promiscuously (UmbA4–6)^16^. Toxin domains are labeled according to their predicted activity or structure: Deam, deaminase; 4TM, four transmembrane helices; LII Phos, lipid II phosphatase. Colors are consistent with depictions of these molecules in other figures. B) Schematic representing the bacterial cell co-precipitation assay to identify *S. griseus*-interaction proteins in sup^Sc^. C) LC-MS-MS-based comparison of protein abundance across two independent replicate samples of *S. coelicolor ∆umbC1–3*-derived sup^Sc^ (“Reference” samples; SC, spectral counts). Spots representing UmbA and UmbB proteins are colored as shown in panel (A). D, H, and J) Comparison of the proportion of spectral counts represented by UmbA and UmbB proteins in reference and cell-associated samples of *S. griseus* (D), *S. achromogenes* (H), *and S. ambofaciens* (J) mixed with *S. coelicolor ∆umbC1–3*-derived sup^Sc^ (LOD, limit of detection). Data represent means from two biological replicates. See panel (B) for experimental setup. E) Growth yields of *S.* *griseus* (measured as relative luminescence units (r.l.u.)) after 16 h of treatment with sup^Sc^ derived from the indicated strains of *S. coelicolor*. Data represent the mean ± s.d. (*n* = 6). F) Outcome of growth competition assays between the indicated strains of *S. coelicolor* (Sc) and *S. griseus* (Sg). Data represent the mean ± s.d. (*n* = 3). Asterisks indicate competitive indices significantly different from wild-type (p<0.0001, one-way ANOVA with log transformed data, Dunnett’s multiple comparisons test). G and I) Growth yields of *S.* *achromogenes* (G) and *S. ambofaciens* (I) after 16 h of treatment with sup^Sc^ derived from the indicated strain of *S. coelicolor*. Data represent means ± s.d. (*n* = 3). See also Table S1.

We recently reported the discovery that *Streptomyces coelicolor* elaborates complex, proteinaceous toxigenic particles that mediate competition with other *Streptomyces* species^16^. Based on their unprecedented morphology, which consists of an extended stalk topped by five spokes, we named these particles umbrella toxins. *S. coelicolor* secretes three distinct umbrella toxin particles composed of proteins belonging to at least three families, UmbA-C^16^. The core of each particle consists of a unique UmbC family member, which harbors the polymorphic toxin domain that confers toxicity. UmbC proteins consist of a ring formed by eight degenerate repeats on one end, connected via a coiled-coil to their toxin domain located at the other end of the assembly. In contrast to the other regions of the proteins, the toxin domains of the three UmbC proteins of *S. coelicolor* bear no recognizable sequence or predicted structural similarity, and correspondingly act via different mechanisms to intoxicate target cells. Five repeats on each UmbC ring are bound by five protomers of a cognate UmbB family protein. In turn, this adaptor protein recruits UmbA family proteins, rooting the five-spoked morphology of the particles. UmbA proteins contain two domains: an N-terminal trypsin-like domain that mediates UmbB binding and a C-terminal predicted lectin domain. While the trypsin-like domains of UmbA proteins are relatively conserved, their predicted lectin domains are highly variable, suggesting they may bind distinct carbohydrate structures. *S. coelicolor* possesses six UmbA proteins (UmbA1–6); UmbA1–3 associate solely with their respective Umb particles, whereas the others, encoded at orphan *umbA* loci, bind promiscuously to all three particles (Figure 1A).

The architecture and compositional heterogeneity of the *S. coelicolor* umbrella particles are unprecedented among characterized interbacterial toxin systems. Indeed, the combinatorial diversity of UmbA proteins allows each particle to adopt up to 1024 structural and 56 compositional variants. This is further augmented by the release of multiple particles bearing distinct toxin domains and a partially overlapping set of UmbA proteins. Here, we show that UmbA proteins mediate receptor binding and particle recruitment to target *Streptomyces* species. We identify a target cell receptor as a previously undescribed glycoconjugate consisting of a teichuronic acid-like (TUA) polymer appended to wall teichoic acids (WTA) and use cryo-EM to elucidate the structural basis for specific recognition by a UmbA lectin. We show that variation in TUA structure across species mediates differential targeting by umbrella toxins, and provide bioinformatic evidence of a molecular arms race driving this diversification and that of umbrella toxin lectins. Our data support a model whereby the variable lectin and toxin domains within cocktails of secreted umbrella particles facilitate a two-tiered bet hedging strategy for interspecies antagonism.

## Results

### UmbA proteins specifically recognize target cells

We previously showed that the Umb particles of *S. coelicolor* act in a highly selective manner to intoxicate other *Streptomyces* spp.^16^ We hypothesized that this selectivity derives from the specific recognition and binding of the variable lectin domains of UmbA proteins – positioned at the tips of the five spokes of Umb particles – to receptor molecules on target cells (Figure 1A). To test this, we sought to measure the capacity of individual *S. coelicolor* UmbA proteins to mediate target cell binding. Prior work by our laboratory demonstrated that the Umb2 particle of *S. coelicolor* is responsible for the inhibitory activity of high molecular weight protein -enriched *S. coelicolor* supernatant (sup^Sc^) against *S. griseus*^16^. Given that *S. coelicolor* Umb particles possess a heterogenous assortment of UmbA proteins (e.g. Umb2 contains UmbA2 and UmbA4–6), assessing whole particle binding was deemed an impractical solution. To circumvent this limitation, we used sup^Sc^ from a strain lacking UmbC1–3, in which UmbA proteins are untethered from each other, as a source of the UmbA proteins (Figure 1B).

Semi-quantitative mass spectrometry showed high reproducibility in the levels of UmbA proteins across biological replicate samples of *S. coelicolor* Δ*umbC1–3*-derived sup^Sc^ (Figure 1C). Next, we applied these samples to *S. griseus* target cells and evaluated the retention of individual UmbA proteins following washing (Figure 1B). Interestingly, among the UmbA proteins, only UmbA4 was detected in association with *S. griseus* (Figure 1D). As expected based on the promiscuity in UmbB binding by orphan UmbA proteins such as UmbA4, we also found UmbB1–3 enriched in the *S. griseus*-associated proteome.

The involvement of UmbA proteins in umbrella toxin function has not been determined. To explore this question, we generated *S. coelicolor* strains bearing in-frame deletions in each of the *umbA* genes and applied sup^Sc^ derived from these to *S. griseus*. Remarkably, similar to the deletion of *umbC2*, inactivation of *umbC4* was sufficient to abrogate the inhibitory activity of sup^Sc^ and to eliminate the fitness benefit to *S. coelicolor* conferred by the Umb2 particle in co-culture with *S. griseus* (Figure 1E,F)^16^. Together, these data suggest that UmbA4 is responsible for specific recognition of *S. griseus* and that this interaction is required for Umb-based intoxication.

Our prior MS data suggest umbrella toxin particles, including Umb2, carry multiple UmbA proteins^16^. Taken together with our current findings, we posited that the UmbA proteins associated with a given UmbC (via UmbB) can recruit the toxin to a multitude of target cells. Testing this necessitated screening a collection of diverse *Streptomyces* spp., which identified *S. achromogens* as an additional target of the UmbC2 toxin (Table S1, Figure 1G). Unlike *S. griseus*, which exclusively bound UmbA4, we found *S. achromogenes* specifically associates with UmbA5 (Figure 1H). Furthermore, among all *umbA* deletion strains, only Δ*umbA5* – not ∆*umbA4* – lost the capacity to intoxicate *S. achromogenes* (Figure 1G). We conclude from these data that a single UmbC can be recruited to different target cells by different UmbA proteins.

UmbA4 and UmbA5 are both orphan UmbA proteins that associate with each of the three Umb particles of *S. coelicolor* (Figure 1A), yet only UmbC2 mediates Umb-based intoxication of *S. griseus* and *S. achromogenes*. One explanation for this phenomenon is that only UmbC2 is active. However, we found that *S. ambofaciens* inhibition by sup^Sc^ occurs independently of UmbC2, and instead requires UmbC1(Figure 1I). Analysis of UmbA binding to *S. ambofaciens* showed both UmbA1 and UmbA6, the latter an orphan UmbA, are retained in complex with *S. ambofaciens* cells (Figure 1J). Interestingly, sup^Sc^ from *S. coelicolor* strains lacking either *umbA1* or *umbA6* retained strong inhibitory activity comparable to that from the wild-type strain. However, combined deletion of *umbA1* and *umbA6* abrogated toxicity, suggesting that either UmbA1 or UmbA6 is sufficient to mediate Umb-based intoxication of *S. ambofaciens* (Figure 1I). This apparent redundancy likely derives from the close relatedness of the lectin domains of UmbA1 and UmbA6, which share 87% amino acid sequence identity, compared to 16% average pairwise identity for the other *S. coelicolor* UmbA lectin domains.

Our finding that multiple species (*S. griseus*, *S. achromogens*, and *S. ambofaciens*) bound by orphan UmbA proteins are intoxicated by a single – and different – UmbC suggests that targets display differential susceptibility to the toxin domains of the umbrella particles produced by *S. coelicolor*. We propose a model in which umbrella toxin particles provide *S. coelicolor* bet hedging against competing species at two levels: (*i*) target cell recognition at the level of UmbA diversity on single particles and (*ii*) target cell intoxication at the level of multiple UmbC proteins with divergent toxin domains recruited by orphan UmbA proteins (Figure 1A).

### A previously undescribed teichuronic acid-like cell surface glycopolymer acts as an umbrella toxin receptor

**Figure 2.**
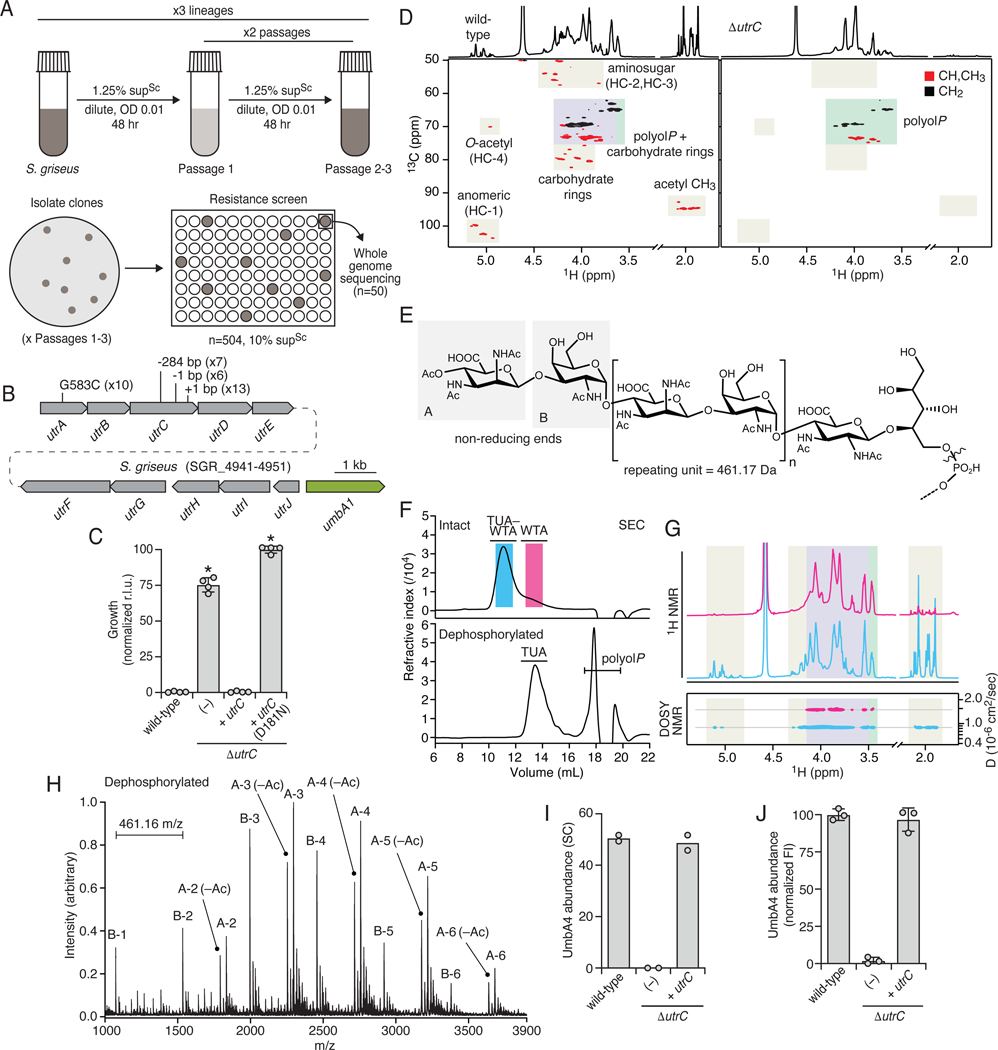
A TUA–WTA hybrid polymer produced by *S. griseus* is required for targeting by *S. coelicolor* umbrella toxins. A) Overview of adaptive laboratory evolution (ALE) experiment to isolate *S. griseus* clones resistant to *S. coelicolor* umbrella toxins. B) Schematic depicting the location of mutations in an *S. griseus* carbohydrate biosynthesis gene cluster selected during ALE with sup^Sc^ treatment. Numbers indicate the number of times the indicated mutation was observed across 50 clones sequenced. The location of the sole *umbA* gene in *S. griseus* is also indicated. C) Growth yields of the indicated strains of *S.* *griseus* (measured as relative luminescence units (r.l.u.)) after 16 h of treatment with sup^Sc^. Data represent the mean ± s.d. (n=4), and Asterisk indicates growth significantly different from the wild-type treatment (p<0.0001, one-way ANOVA, Dunnett’s multiple comparisons test). D) Partial views of 2D multiplicity-edited ^1^H-^13^C HSQC NMR spectra of *S. griseus* wild-type (left) and Δ*utrC* (right) intact WTA polymers obtained by hydrolysis from isolated cell wall sacculi. The corresponding 1D ^1^H spectra are shown above each HSQC panel. Grey shading indicates peaks deriving from carbohydrates; green shading indicates poloyl*P*-derived peaks; blue shading indicates peaks deriving from both polyol*P* moieties and carbohydrates. E) Structure determined for the *S. griseus* TUA →[4)-ManNAc3NAcA-β(1→3)-GalNAc-α(1→]_n_ oligomer. ~70% of the TUA is terminated with a 4OAc-ManNAc3NAcA residue (non-reducing end A), while ~30% is terminated with a GalNAc residue (non-reducing end B). The reducing-end residue of GlcNAc3NAcA forms a β(1→4) glycosidic bond with a ribitol-5-phosphate residue. The dashed bond indicates the expected linkage with the polyol*P* WTA polymer; the wavy line indicates the site of cleavage upon chemical dephosphorylation. F) SEC analysis of intact (top) or dephosphorylated polymer obtained from *S. griseus* wild-type cell wall preparations by TCA hydrolysis. G) ^1^H NMR (top) and DOSY (bottom) spectra of material from the two SEC peaks obtained for the total cell wall polymer isolate. NMR peak colors correspond to panel (F), shading corresponds to panel (D). D, diffusion coefficient. H) MALDI-TOF mass spectrometry analysis of dephosphorylated polymer obtained from wild-type *S. griseus.* Masses corresponding to [M−H]^−^ ions of TUA oligomers with reducing ends A (2–6 repeating units) and B (1–6 repeating units) were detected. The signals of the acetylated reducing end A oligomers were accompanied by secondary peaks due to the loss of the acetyl group. I) UmbA4 abundance following cell co-precipitation with the indicated strains of *S. griseus*. Data represent means from two biological replicates. J) UmbA4 abundance by fluorescence intensity (FI) following incubation of TRITC-labeled UmbA4 protein with the indicated strains of *S. griseus*. Data represent the mean ± s.d. (n=3). See also Figure S1, S2, and Table S2–4.

If UmbA proteins bind target cells selectively, variable target cell surface molecules should serve as umbrella particle receptors. To identify candidate umbrella particle receptors, we selected for *S. griseus* strains resistant to intoxication by sup^Sc^ (Figure 2A). After three passages at an intermediate concentration of sup^Sc^, multiple lineages of *S. griseus* acquired resistance to the treatment (Figure S1A). Analysis of individual clones isolated from each passage revealed that only 6% (28/432) of them exhibited sup^Sc^ resistance after one passage, which increased to 35% (17/48) and 100% (24/24) after two and three passages, respectively (Figure S1B). Whole genome sequencing of 50 resistant clones deriving from all passages and lineages revealed 11 genes with mutations in multiple clones (Table S2). Those within a single previously uncharacterized gene cluster, which we named umbrella toxin resistance genes A-E (*utrA*-*E*), were most prevalent (72%) and in many cases accounted for the only non-synonymous mutation in a given clone (Table S2 and Figure 2B). To directly evaluate the role of a *utr* gene in umbrella toxin resistance, we generated a *S. griseus* strain bearing an in-frame deletion of *utrC*. Like strains selected by toxin exposure, the growth of this strain was unaffected by sup^Sc^ at a level sufficient to fully inhibit growth of the wild-type and complementation restored Umb sensitivity (Figure 2C).

UtrC possesses a predicted glycosyltransferase domain distantly resembling that found in *S. aureus* TarS, a protein that installs β-linked N-acetylglucosamine on the ribitol phosphate backbone of wall teichoic acids (WTAs) (Figure S1C, Table S3)^17,18^. This domain includes a predicted catalytic aspartate and we found that substitution of the corresponding residue in UtrC with asparagine (D181N) abolishes sup^Sc^ sensitivity (Figure 2C)^17^. Interestingly, further analyses of the *utrA-E* cluster indicated that all five of the genes encode proteins strongly predicted to act on carbohydrates (Figure 2B and Table S3). For example, *utrA*, the other gene hit in our selection, encodes a likely sugar aminotransferase and *utrE* encodes a predicted epimerase. Also of note, immediately downstream of this gene cluster is a divergently transcribed operon of five genes, which we named *utrF-J*, encoding additional predicted carbohydrate-modifying enzymes. One of these, UtrF shows strong predicted structural similarity to *S. aureus* TarM, which installs β-linked N-acetylglucosamine on the ribitol phosphate backbone of WTAs^19,20^. Adjacent to *utrJ* is the ORF encoding the sole *S. griseus* UmbA ortholog, further suggesting a link between the *utr* genes and umbrella toxin-based antagonism.

Given the bioinformatic links between multiple Utr proteins and WTA biosynthetic enzymes, we sought to compare WTA structures of *S. griseus* wild-type and Δ*utrC* strains. WTAs from these strains were obtained by hydrolysis from isolated cell wall sacculi as described previously with minor modifications^21^. Analysis of NMR spectra of the wild-type preparation revealed ^1^H-^13^C HSQC peaks corresponding to carbohydrate and polyol phosphate (polyol*P*) moieties, consistent with carbohydrate-modified WTA structures present in related organisms (Figure 2D)^22^. Strikingly, while the ∆*utrC* preparation contained similar polyol*P* peaks, those attributable to carbohydrates were either strongly diminished or undetected. This finding suggests that the glycosyltransferase encoded by *utrC* is required for the biogenesis of the predominant carbohydrate modification to the WTA of *S. griseus*.

Detailed analyses of our 2D NMR data revealed that the carbohydrate molecule present in wild-type samples and missing from ∆*utrC* consists of an extended glycan polymer with a repeating unit of →3)GalNAc-α(1→4)-ManNAc3NAcA-β(1→, alternately terminated at the non-reducing end by 4-OAc-ManNAc3NAcA or GalNAc residues (Figure 2E, Figure S2A and Table S4). The repeating portion of this structure is reminiscent of teichuronic acids (TUAs), a distinct and poorly characterized class of cell wall-associated anionic polymers found in *Streptomyces* and several other genera of Actinomycetota and Bacillota^22,23^.

TUAs are tacitly defined by the presence of a uronic acid that forms 2–4-membered repeating units with neutral monosaccharides^23^. In certain species, including *Micrococcus luteus* and *Bacillus licheniformis*, there is evidence for direct linkage of TUAs to the cell wall by a phosphodiester bond to the *N*-acetylmuramic acid sugar of peptidoglycan^24,25^. However, our analyses indicate that in the *S. griseus* TUA-like molecule we identified, the reducing-end sugar, GlcNAc3NAcA, is directly linked by a β glycosidic bond to the 4-hydroxyl of ribitol 5-phosphate (Figure 2E, Figure S2A,D, and Table S4). Peaks in the ^1^H-^13^C HSQC spectra not attributable to this TUA-like molecule derive from 1,5- and 1,3,5-linked ribitol*P* units, suggestive of branching ribitol WTA, and a terminal 3-linked glycerol*P* moiety, indicative of glycerol substitution in the polymer^26,27^. In contrast, spectra from the equivalent polymer from *S. griseus* ∆*utrC* suggest it lacks the branching-associated linkage (1,3,5-linked ribitol*P*), and contains an additional terminal 5-linked ribitol*P* residue. We propose this latter residue replaces the 5-linked ribitol*P* that is substituted with the TUA-like molecule in wild-type *S. griseus*. These findings raised the intriguing possibility that in *S. griseus*, and perhaps other *Streptomyces* species, TUA polymers are covalently linked to WTAs and that this hybrid molecule could serve as a receptor for umbrella toxins.

To begin evaluating these possibilities, we performed size exclusion chromatography on the material released from a wild-type *S. griseus* cell wall preparation. Analysis of fractions representing the two resulting peaks by ^1^H and ^1^H-^13^C HSQC NMR provided evidence that the higher mass species constitutes a polymer with both the TUA-like and WTA components, whereas the lower mass peak contains only those indicative of WTAs (Figure 2F, Figure S2B and Table S4). Diffusion-ordered NMR spectroscopy (DOSY) showed that the *S. griseus* TUA and WTA polymers have the same hydrodynamic properties in the higher-mass species, indicative of a single molecule (Figure 2G). The complexity of ^1^H, ^13^C and ^31^P spectra prevented establishing a direct link between the TUA and WTA by NMR. However, dephosphorylation of the material yielded a major species with significantly reduced molecular weight, consistent with degradation of the polyol*P* WTA backbone (Figure 2F). Analysis of the dephosphorylated material by NMR and mass spectrometry confirmed the residual structure as that of the intact TUA portion of the glycoconjugate (Figure 2H, Figure S2C and Table S4). Together, these data indicate that UtrC is required for biosynthesis of an extended, TUA-like polymer linked to a *S. griseus* WTA backbone (Figure S2D,E).

If TUAs are a receptor for the Umb2 particle of *S. coelicolor*, we reasoned that the interaction of UmbA4 with *S. griseus* should require their presence on the cell surface. Indeed, we found that UmbA4 is not retained on the surface of *S. griseus* cells lacking UtrC, and that UmbA4 interaction is restored by expression of UtrC from an ectopic locus (Figure 2I). UmbA4 purified from *S. coelicolor* Δ*umbC1–3*-derived sup^Sc^ also associated with wild-type, but not ∆*utrC S. griseus* cells, indicative of a direct interaction between the molecules (Figure 2J). These data show that, via UmbA4, *S. coelicolor* exploits TUA as a receptor for Umb2 particle targeting.

### Diversity of *Streptomyces* TUA biosynthetic gene clusters

**Figure 3.**
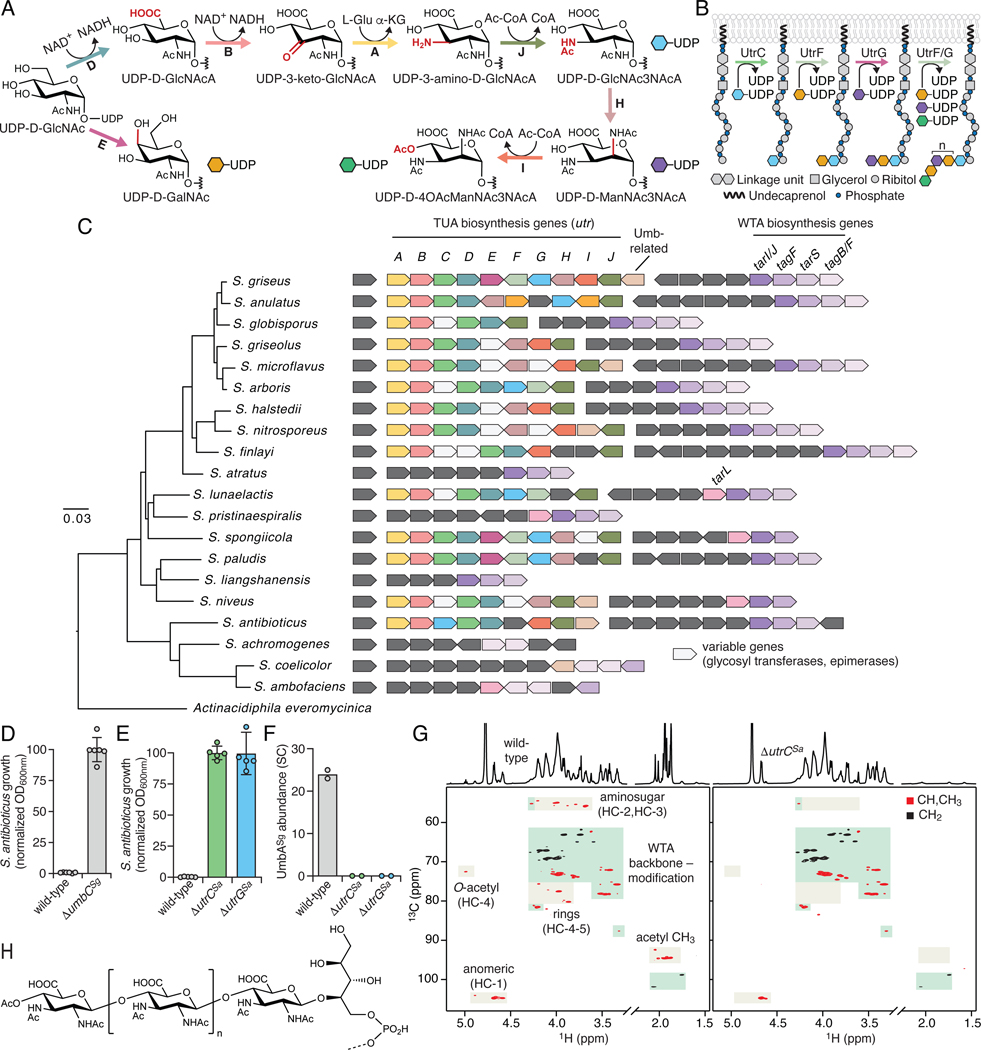
*Streptomyces* TUA biosynthetic loci are variable and produce distinct carbohydrate structures. A, B) Predicted pathway for the biosynthesis of TUA by the Utr pathway of *S. griseus*. Key changes for each step in sugar precursor biosynthesis (A) are highlighted in red, and arrows representing the enzymes responsible for each reaction are colored according to panel (C). Colors of TUA monosaccharides and biosynthetic enzymes involved in polymer generation on WTA ribitol phosphate (B) correspond to panels (A) and (C), respectively. C) Phylogeny (derived from the bacterial Genome Taxonomy Database (GTDB)) of selected *Streptomyces* species indicating gene content of TUA and linked WTA biosynthetic loci. Shared coloring indicates orthologous genes involved in TUA or WTA biosynthesis; all genes encoding umbrella toxin particle subunits are colored the same, regardless of orthology. Candidate TUA biosynthetic genes lacking orthologs in *S. griseus* are indicated in light grey and dark grey indicates genes with unrelated functions. Genes not drawn to scale. D) Growth yields of *S. antibioticus* as measured by optical density (OD_600nm_) after 16 h of treatment with sup^Sg^ derived from the indicated strains of *S. griseus*. Data represent means ± s.d. (*n* = 6). E) Growth of the indicated strains of *S. antibioticus* after 16 h of treatment with sup^Sg^ derived from *S. griseus* wild-type strain. Data represent means ± s.d. (*n* = 5). F) UmbA^Sg^ abundance in cell-associated samples following precipitation from *S. griseus* ∆*umbC*-derived sup^Sg^ with *S. antibioticus* cells of the indicated genetic backgrounds. Data represent means from two biological replicates. G) Partial views of 2D multiplicity-edited ^1^H-^13^C HSQC NMR spectra of *S. antibioticus* wild-type (left) and Δ*utrC* (right) intact WTA polymers obtained by hydrolysis from isolated cell wall sacculi. The corresponding 1D ^1^H spectra are shown above each HSQC panel. Grey shading indicates peaks deriving from carbohydrates; green shading indicates poloyl*P*-derived peaks. H) Structure of the *S. antibioticus* TUA oligomer. The dashed bond indicates the expected linkage with the polyol*P* WTA polymer. See also Figure S3, S4, and Table S1, S2, S4.

Our finding that variable UmbA lectin domains dictate the specificity of umbrella toxin targeting implies that the glycopolymers they target must also be diversified. We hypothesized that an evolutionary arms race between umbrella toxin producers and their target organisms – a dynamic in which the evolution of escape mutants in one population would select for compensatory toxin alterations in the second – could be a driver of TUA structural diversification in parallel with the extensive UmbA diversity we previously identified^16^. To assess the extent to which TUA structure can vary, we first determined a plausible TUA biosynthetic pathway. The proteins encoded in the Utr locus bear no significant resemblance to the TUA biosynthetic enzymes of *Bacillus subtilis,* indicating *Streptomyces* employ an alternate route to the biosynthesis of the molecule^28^. Nevertheless, using structural modeling-based predictions, we were able to assign a Utr protein to each step of a credible biosynthetic pathway for TUA (Figure 2B, Figure 3A,B and Table S3).

Our analysis revealed that the *S. griseus* TUA precursor UDP-ManNAc3NAcA is likely produced from UDP-GlcNAc by a similar pathway to that found in *Pseudomonas aeruginosa*, which incorporates ManNAc3NAcA into lipopolysaccharide (Figure 3A, Figure 2B and Table S3)^29^. Enzymes involved in this pathway include a UDP-sugar dehydrogenase (*utrD*), a sugar amino transferase and oxidase pair (*utrA, utrB*), an N-acetyltransferase (*utrJ*), and a UDP-glucose 2-epimerase (*utrH*). The *S. griseus* TUA gene cluster additionally encodes a UDP-glucose 4-epimerase, *utrE*, which we predict is responsible for generating UDP-GalNAc, the second sugar of the repeating unit, from UDP-GlcNAc.

The remaining Utr proteins are predicted to participate in polymer assembly or modification (Figure 3B, Figure 2B and Table S3). UtrC and UtrF bear closest structural resemblance to TarS and TarM, respectively, leading us to predict that UtrC links GlcNAc3NAcA to ribitol phosphate and UtrF performs the subsequent installation of α(1→4) linked GalNAc (Figure S1C)^17,19^. We predict the repeated disaccharide portion of the TUA polymer of *S. griseus* is generated by alternating UtrG-catalyzed addition of β(1→3) linked ManNAc3NAcA and further installation of α(1→4) linked GalNAc by UtrF. The final step in the pathway, *O*-acetylation of the non-reducing end sugar, is likely performed by the remaining protein encoded in the locus, the predicted acetyltransferase UtrI.

With a plausible route to *S. griseus* TUA biosynthesis established, we searched for evidence of predicted TUA structural diversity within the respective biosynthetic loci of 15 closely related species. For reference, we also included a small group of more distantly related umbrella toxin target species and *S. coelicolor*. Outside of a handful of core genes, we find considerable variability in the genes present in TUA biosynthetic loci (Figure 3C). For example, the TUA gene cluster of *S. anulatus*, which is not a target of *S. coelicolor* umbrella toxins^16^, lacks predicted epimerase and *O*-acetyl-transferase genes found in its close relative *S. griseus*. Other epimerases, acetyltransferases and glycosyltransferases encoded within the TUA loci varied extensively, suggesting the number and type of monosaccharides present in the repeating unit and the decorations found at the non-reducing end vary across species.

A subset of the species we examined appear to lack TUA biosynthetic capacity entirely. Interestingly, species lacking TUA biosynthesis genes include two identified targets of *S. coelicolor* umbrella toxins, *S. ambofaciens* and *S. achromogenes* (our previous study^16^ and Figure 1G), suggesting UmbA lectin domains may recognize additional cell surface carbohydrates beyond the hybrid TUA–WTA polymers identified here (Figure 3C). These genomic analyses provide evidence of selection for diversification in the glycopolymers presented on the *Streptomyces* cell surface, as would be predicted to occur in an umbrella toxin-mediated arms race scenario.

### A distinct TUA structure mediates targeting by a divergent UmbA protein

Though itself a target of *S. coelicolor* Umb2, *S. griseus* possesses the genetic elements we posited encode the necessary subunits of a minimal active umbrella particle: a single UmbA (UmbA^Sg^), UmbB (UmbB^Sg^), and a toxin-domain containing UmbC (UmbC^Sg^) (Figure S3A,B). Additionally, immediately downstream of *umbC*^*Sg*^ is a gene encoding the predicted immunity factor for the toxin, UmbD^Sg^. Thus, to investigate the function of umbrella toxins outside of *S. coelicolor*, we generated an in-frame deletion in *S. griseus umbC* and compared the capacity of high molecular weight protein-enriched *S. griseus* supernatant (sup^Sg^) from this strain versus the wild-type to inhibit a panel of diverse *Streptomyces* spp. Similar to the behavior of sup^Sc^, we found that sup^Sg^ potently and selectively inhibits the growth of other *Streptomyces* spp. in a *umbC*-dependent manner. Of the 37 strains included in this screen, we identified *S. antbioticus* as an apparent target of the *S. griseus* umbrella toxin (Figure S3C, Table S1 and Figure 3D)^30^.

To explore the generality of our findings pertaining to the significance of target cell surface carbohydrate recognition by UmbA proteins, we subjected *S. antibioticus* to selection in the presence of sup^Sg^. After five passages, we screened 96 isolated clones and confirmed eight to be resistant to sup^Sg^ (Figure S3D). Whole genome sequencing of six resistant isolates revealed non-synonymous mutations in *utrC* and a gene encoding a second glycosyltransferase in the TUA operon, *utrG*, in multiple strains (Table S2, Figure 3C and Figure S3E). In-frame deletions in these genes confirmed that their inactivation grants resistance to sup^Sg^ and abrogates UmbA^Sg^ binding to *S. antibioticus* (Figure 3E,F).

The TUA gene clusters of *S. griseus* and *S. antibioticus* show substantial differences (Figure 3C). For example, the *S. antibioticus* gene cluster lacks genes encoding two predicted epimerases and a glycosyltransferase found in *S. griseus* (*utrE, utrF* and *utrH*). We reasoned that these differences in gene content between the clusters manifest in structural differences in their TUAs, potentially explaining how *S. griseus* avoids umbrella particle adsorption to its own cell surface and why *S. coelicolor* umbrella particles do not target *S. antibioticus*^16^. To test this, we determined the structure of the major species within material recovered from purified cell wall sacculi of *S. antibioticus* wild-type and *∆utrC* following TCA hydrolysis. NMR spectra indicated the presence of carbohydrate residues consistent with TUA only in wild-type-derived material, whereas both samples possessed apparent WTA polyol*P* residues (Figure 3G, Figure S4A,B,D and Table S4). DOSY analysis of the material revealed peaks attributable to TUA and WTA exhibit identical hydrodynamic properties, as observed in *S. griseus* (Figure S4E). Detailed analyses showed that the TUA polymer of *S. antibioticus* consists of β−1,4-linked GlcNAc3NAcA monosaccharide residues, rather than the repeating disaccharide unit found in *S. griseus* (Figure 3G,H, Figure S4A,C and Table S4). This lends support to our proposed biosynthetic pathway for TUA, as the enzyme we predict is responsible for adding the second monosaccharide of the repeating unit in *S. griseus*, UtrF, is absent from *S. antibioticus* (Figure 3C). Acetylation at the 4-hydroxy position of the non-reducing end sugar is a common feature of the TUA structures, consistent with both species encoding the predicted acetyltransferase UtrI. Also as in *S. griseus,* the reducing-end GlcNAc3NAcA in the TUA-like structure forms a β−1,4 glycosidic bond with a ribitol-5*P* residue, and the WTA portion of the TUA–WTA hybrid molecule of *S. antibioticus* is composed of ribitol phosphate. In the latter, the 1,5-linked ribitol*P* is heavily decorated with glucose, a modification not observed in *S. griseus* (Figure S4D). Additional *S. antibioticus* WTA substitutions include lysine and phosphoethanolamine. We conclude that TUA–WTA hybrid molecules are found in *Streptomyces* beyond *S. griseus* and that differences in TUA structure across species determine umbrella toxin susceptibility.

### Retargeting of umbrella particles using lectin domain swapping

**Figure 4.**
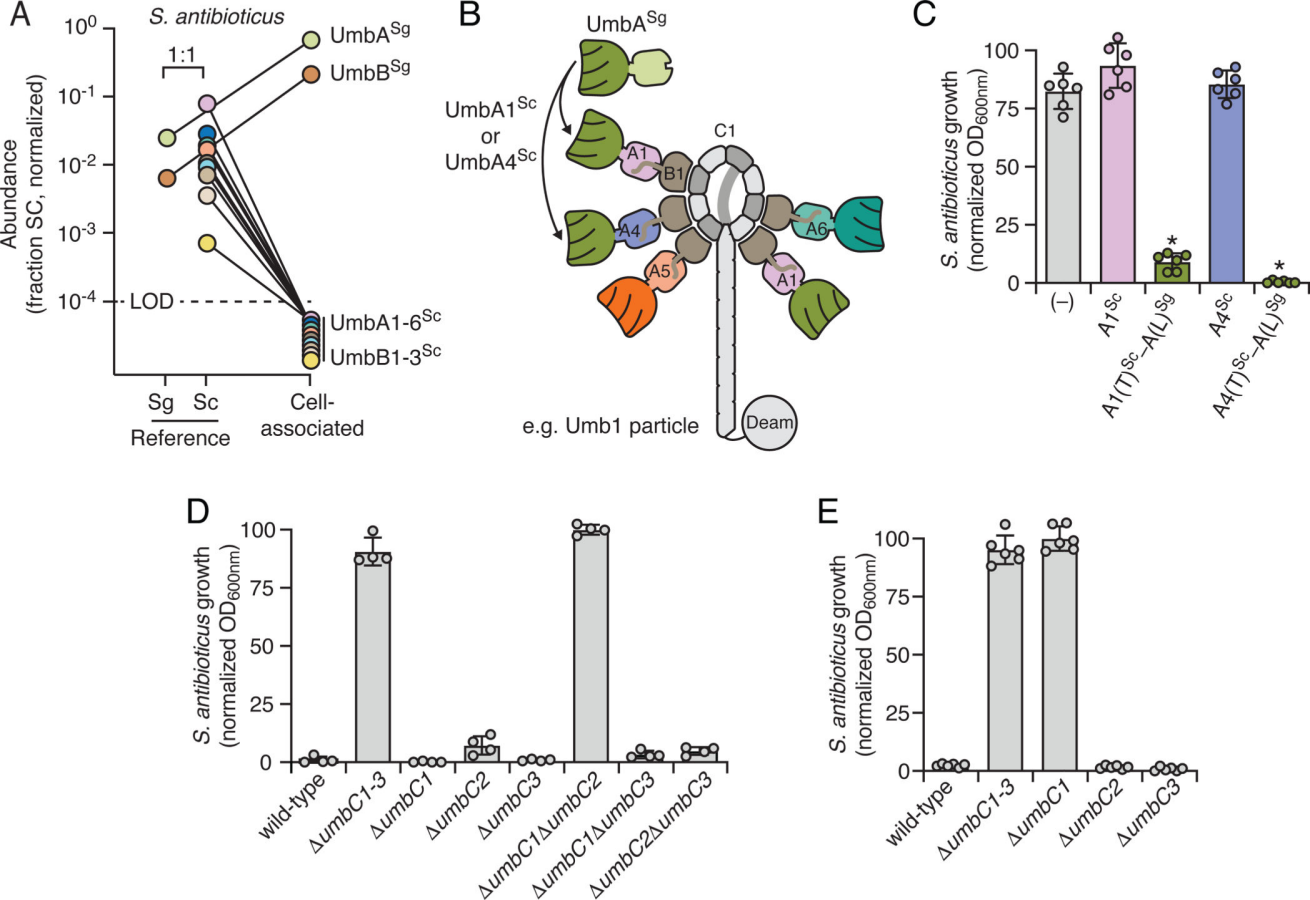
Swapping *S. coelicolor* lectin domains with that of *S. griseus* expands umbrella particle target range. A) Comparison of the proportion of spectral counts (SC) represented by UmbA and UmbB proteins in reference and cell-associated samples derived from treatment of *S. antibioticus* cells with a 1:1 mixture of *S. coelicolor ∆umbC1–3*-derived sup^Sc^ and *S. griseus ∆umbC*-derived sup^Sg^ (limit of detection, LOD). For the reference samples, proteins derived from *S. griseus* and *S. coelicolor* are indicated below for clarity. B) Schematic illustrating the makeup of the *S. coelicolor* Umb1 particle containing chimeric UmbA proteins. Chimeric UmbAs consist of the N-terminal trypsin-like domain of UmbA1^Sc^ or UmbA4^Sc^ fused to the lectin domain of UmbA^Sg^. C) Growth as measured by optical density (OD_600nm_) of *S. antibioticus* after 16 h of treatment with sup^Sc^ derived from *S. coelicolor* expressing the indicated native or chimeric UmbA. Data represent the mean ± s.d. (*n* = 6). Asterisks indicate competitive indices significantly different from wild-type (p<0.0001, one-way ANOVA with log transformed data, Dunnett’s multiple comparisons test). D) Growth of *S. antibioticus* after 16 h of treatment with sup^Sc^ derived from the indicated strain of *S. coelicolor* expressing the chimeric protein UmbA4(T)^Sc^- A(L)^Sg^. Data represent the mean ± s.d. (*n* = 4). E) Growth of *S. antibioticus* after 16 h of treatment with sup^Sc^ derived from the indicated strain of *S. coelicolor* expressing the chimeric protein UmbA1(T)^Sc^- A(L)^Sg^. Data represent the mean ± s.d. (*n* = 6).

The modularity of UmbA proteins motivated us to ask whether their lectin domains could be exchanged to retarget an umbrella toxin particle. The C-terminal β-propeller lectin domain of UmbA^Sg^ diverges significantly from the lectin domains of *S. coelicolor* UmbA1–6 (20–30% identity). We reasoned that could explain why the single umbrella particle elaborated by *S. griseus* is able to target *S. antibioticus*, whereas none of the three produced by *S. coelicolor* are^16^. To test this, we prepared a mixture of sup^Sg^ and sup^Sc^, applied this to *S. antibioticus*, and used MS to evaluate the retention of Umb proteins after washing. Despite the multitude of UmbA proteins in sup^Sc^, we detected only UmbA^Sg^ and UmbB^Sg^ in association with *S. antibioticus* (Figure 4A). Given this, we attempted to synthetically retarget umbrella particles by replacing the lectin domains of *S. coelicolor* UmbA1 and UmbA4 proteins with that of UmbA^Sg^ (Figure 4B). Remarkably, we found that sup^Sc^ deriving from both of the chimeric UmbA-expressing strains strongly inhibits *S. antibioticus* growth. Inhibition was not observed in cultures treated with sup^Sc^ from control cells expressing the native UmbA proteins (Figure 4C). These data demonstrate that the lectin domains of UmbA proteins are sufficient to confer umbrella particle target cell specificity.

Next, we used this synthetic system to further probe our model for UmbA recruitment of the three umbrella particles and their accompanying UmbC-linked polymorphic toxins. Using sup^Sc^ prepared from *S. coelicolor* strains expressing chimeric UmbA4 and bearing mutations in individual or combinations of the *umbC1–3* genes, we found that *S. antibioticus* growth is effectively inhibited by both UmbC1 and UmbC2 (Figure 4D). This contrasts with the native umbrella toxin target of *S. coelicolor*, *S. griseus*, which when bound by UmbA4 is inhibited exclusively by UmbC2. As expected, only the deletion of *umbC1* impacted the *S. antibioticus* growth inhibitory effect of sup^Sc^ derived from the UmbA1 chimeric strain (Figure 4E). The variable efficacy of umbrella toxins against different species supports our hypothesis that releasing umbrella particles with distinct toxins and an overlapping set of receptor binding proteins provides an additional level of bet hedging.

### Two sites on the UmbA4 β-propeller recognize distinct TUA moieties

While our genetic and biochemical analyses establish that lectin diversity underlies species-specific targeting, the molecular basis for how these domains discriminate among TUA structures to achieve this specificity remained unclear. To address this, we determined a cryo-EM structure of *S. coelicolor* UmbA4 bound to purified *S. griseus* TUA oligosaccharide. Given the marked compositional heterogeneity of intact umbrella particles and the challenges associated with recombinantly producing UmbA4, we purified secreted UmbA4–UmbB1–3 complexes (UmbA4 associates with UmbB1–3) from *S. coelicolor* Δ*umbC1–3*. Cryo-EM analysis enabled the determination of a 4.3 Å reconstruction of apo UmbA4 (Figure S5A-E and Table S5).

**Figure 5.**
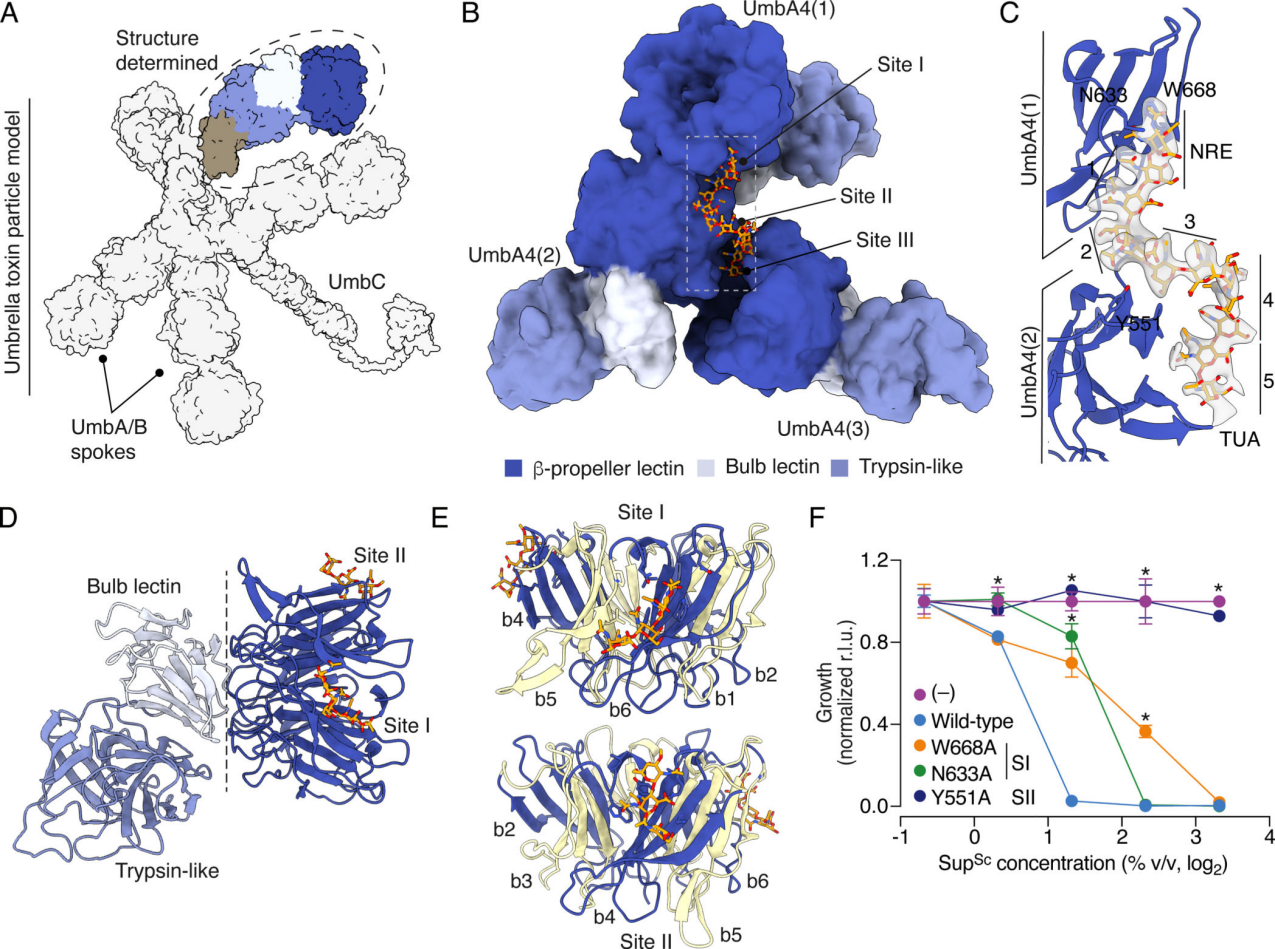
Structure of the UmbA4–TUA complex. A) Schematic depicting the *S. coelicolor* Umb1 particle indicating the localization of the UmbA–UmbB complex investigated here. B) Low pass filtered (6 Å) model derived from our cryo-EM structure of three UmbA4 promoters bound to TUA purified from *S. griseus.* Three distinct sites on the β-propeller domains of different UmbA4 protomers that make contact with TUA are indicated. C) Zoom-in view of UmbA4–TUA interaction sites I and II boxed in panel (B). Side chains for contacting amino acids from different UmbA4 protomers corresponding to those tested in (F) are indicated. Cryo EM density displayed only for TUA; numbers indicate TUA repeating units. NRE, non-reducing end. D, E) Side-on model of a single UmbA4 molecule indicating sites that make productive contact with TUA in different protomers in our cryo-EM structure. Individual blades of the β-propeller are labeled in (E) (b1–6). The TUA position in site I is located between b5 and b6 (top); in site II it is located within b4 (bottom). F) Growth yields of *S. griseus* following 10 h treatment with increasing concentrations of sup^Sc^ derived from *S. coelicolor* strains expressing UmbA4 variants with the indicated amino acid substitutions. UmbA4 levels were normalized across sup^Sc^ samples prior to dilution. Data represent the mean ± s.d. (*n* = 3). Asterisks indicate inhibition by mutant-derived sup^Sc^ significantly different from wild-type (p<0.01, one-way ANOVA with Dunnett’s multiple comparisons test). See also Figure S5, S6, and Table S5.

Addition of *S. griseus* TUA to UmbA4–UmbB1–3 led to formation of helical filaments of ~160 Å in width and >100 nm in length (Figure S5F-G). We therefore applied filament selection and subjected the extracted regions to helical refinement yielding a reconstruction at 3.3 Å resolution (Figure S5H-K, see Methods). UmbA4 consists of three domains, an N-terminal trypsin-like domain, a central bulb lectin, and a C-terminal β-propeller (Figure 1A and Figure 5A), each of which were modeled in the cryo-EM map (Figure 5B). Formation of the helical filaments is largely mediated by the β-propeller domain and the resolution of the reconstruction gradually decreases towards the periphery of the helical assembly. As a result, the UmbB density which protrudes radially from the helix is more weakly resolved, which is further compounded by its compositional heterogeneity, and was therefore not modeled (Figure S5I,J). The map resolves TUA molecules bridging multiple UmbA4 β-propellers, promoting oligosaccharide-induced oligomerization (Figure 5B). The TUA was built into the cryo-EM density beginning at the non-reducing end, the location of which could be deduced based on the termination of density and consideration of the steric restraints imposed by WTA attachment at the reducing end. Since the TUA polymers we purified are variable in length, we extended the TUA chain by cycles of repeating disaccharide addition and refinement until density no longer supported further addition. The final model comprises 12 monosaccharides: the unique non-reducing disaccharide and five repeating disaccharide units (Figure 5B,C and Table S5).

In our structure, a single TUA molecule contacts distinct sites (sites I-III) of the β-propeller domain within three UmbA4 protomers (Figure 5B). Sites I and II occur within the canonical carbohydrate binding pockets of β-propeller lectins (Figure 5D,E). Site III, which we do not consider further, is likely fortuitous, as it is shared between two UmbA protomers and composed of residues outside of typical β-propeller carbohydrate interaction sites (Figure S6A,B). Site I, located between blades 5 and 6, accommodates the non-reducing end 4-OAc-ManNAc3NAcA-β(1→3)-GalNAc disaccharide and uronic acid moiety of the preceding repeat unit (Figure 5C-E). Site II, located on blade 4, interacts with an internal segment of TUA consisting of a repeating unit and a GalNAc residue from an adjacent repeat. In an intact umbrella toxin particle, UmbA4 molecules are tethered to UmbC through interactions with UmbB, positioning them ~100Å apart, making it unlikely that a single TUA molecule would occupy sites I and II on different UmbA4 protomers from the same umbrella particle. It is conceivable that a long TUA molecule could span sites I and II on the same UmbA4; however, given energetic considerations and the size distribution of TUA we observed, we posit that most UmbA4 molecules interact with distinct TUA polymers at each binding site.

Like other β-propellers, the blades of UmbA4 are composed of a degenerate repeating sequence motif (Figure S6C)^31^. Sequences constituting the core fold of the blades and those forming the loops typically involved in Ca^2+^ binding (Ca^2+^ is not resolved in our structure) are highly conserved across blades, whereas the positions forming the carbohydrate binding pockets diverge substantially (Figure S6C). Nevertheless, our model suggests that residues occupying equivalent positions in sites I and II form a subset of the crucial contacts with TUA (Figure 5C, Figure S6D-F). Examples are Trp668 (Site I) and Tyr551 (Site II), which engage TUA through electrostatic interactions. To test the functional significance of these interactions, we generated sup^Sc^ from *S. coelicolor* expressing UmbA4(W668A) or UmbA4(Y551A), normalized these samples to contain equivalent levels of UmbA4, and evaluated the capacity of each to intoxicate *S. griseus*. Remarkably, both single substitutions significantly impaired umbrella toxin activity, with UmbA4(Y551A) reducing it to the limit of detection (Figure 5F). These results motivated us to further probe predicted contacts in site I. Our model suggests that the Asn633 side chain forms polar contacts with the non-reducing TUA sugar (Figure 5C, Figure S6E). Consistent with these interactions, we found sup^Sc^ derived from a strain expressing UmbA4(N633A) exhibits reduced capacity to inhibit *S. griseus* growth (Figure 5F). In total, these structural and physiological data provide a mechanism for the fidelity of UmbA-dependent recruitment of umbrella particles to target cells via TUA interaction.

### Toxin and lectin diversification is a general feature of umbrella toxins in Streptomycetaceae

**Figure 6.**
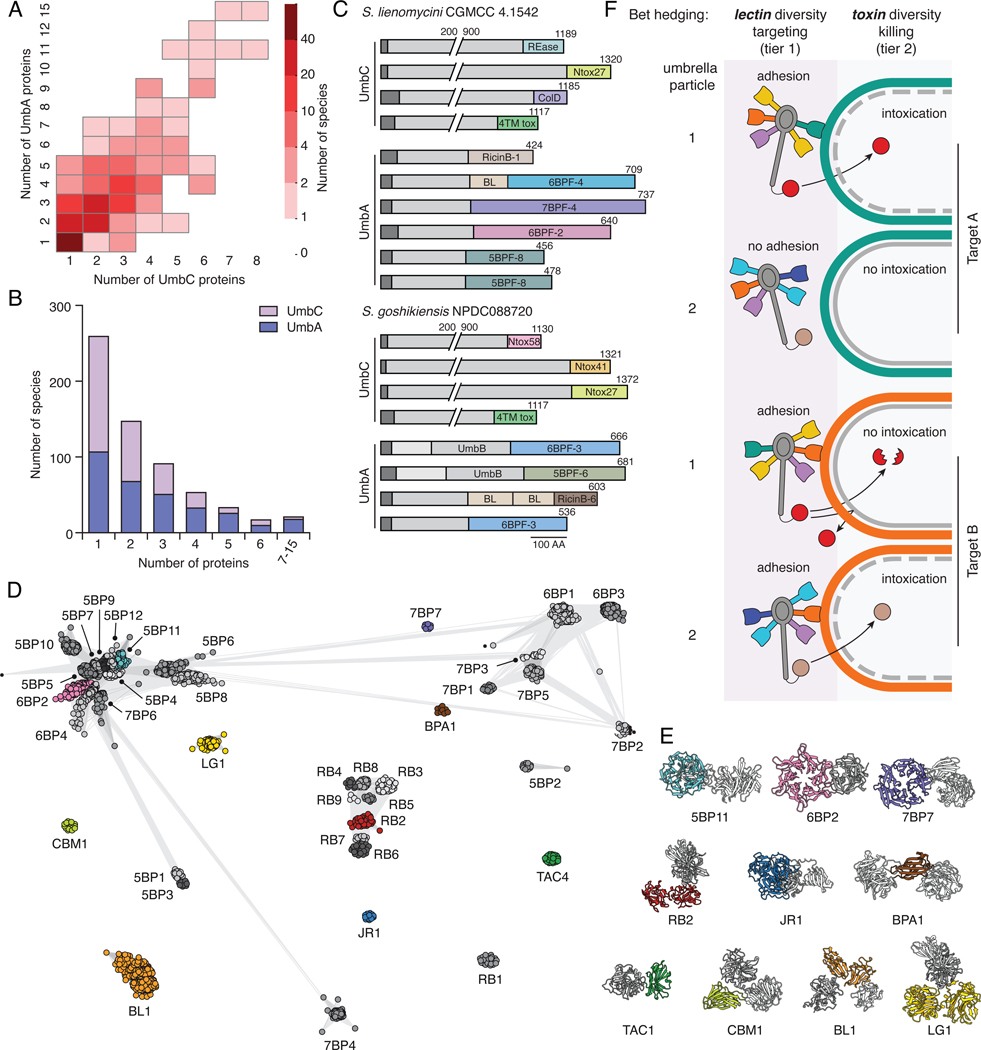
Toxin and lectin multiplicity is a widespread feature of umbrella toxin repertoires across Streptomyceteae. A) Heatmap showing the number of *Streptomyces* species encoding the indicated numbers of UmbA and UmbC proteins. B) Stacked histogram representing the number of *Streptomyces* species encoding the indicated number of UmbA proteins (bottom bar) and UmbC proteins (top bar). C) Schematic illustrating architecture of toxin domains within all UmbCs and lectin domains within all UmbAs encoded by selected *Streptomyces* species *S. lienomycini* CGMCC 4.1542 and *S. goshikiensis* NPDC088720. Secretion signals are indicated in dark grey and length in amino acids is provided. For ease of representation, amino acids 200–900 are not shown for UmbC proteins. REase, restriction endonuclease; ColD, colicin D; 4TM tox, four transmembrane helices toxin; BPF, β-propeller fold; BL: bulb lectin; AA, amino acids. D) Network clustering analysis of UmbA C-terminal domains from diverse *Streptomyces* species. Only those domain families containing at least three family members and predicted to act as lectins are shown for clarity. (BP, β-propeller; RB, ricinB lectin; LG, laminin G; JR, jellyroll-like; BP, BPA-Ig; CB, CBM_4_9; BL, bulb lectin; TAC, TAC4. E) Structural models of UmbA proteins with C-terminal domains representative of different families. Labeled domains are colored according to panel (D). All other UmbA domains depicted in grey. F) Model demonstrating the two-tiered bet hedging strategy employed by bacteria encoding multiple UmbA and UmbC proteins. Potential outcomes of antagonistic interactions are depicted, with umbrella particles 1 and 2 varying in their ability to adhere to and intoxicate targets A and B. Toxin degradation or failure of toxin delivery could prevent intoxication after successful adhesion, among other mechanisms. See also Table S6.

Our experimental data highlight potential evolutionary benefits of the unusual architecture and heterogeneous composition of the *S. coelicolor* umbrella toxin particle cocktail. However, we also investigated an *S. griseus* strain that secretes a single umbrella particle bearing a single UmbA protein. We reasoned that if umbrella toxin particles equipped with multiple UmbA proteins provide a bet hedging advantage that is enhanced by the concurrent release of multiple umbrella particles, we would expect to find *Streptomyces* beyond *S. coelicolor* with this capacity. To evaluate this, we identified UmbA and UmbC-encoding genes among a collection of 11,223 Streptomycetaceae genomes, resulting in a total of 9577 and 7099 sequences, respectively (Table S6). We observed that 66% of species with the capacity to synthesize an umbrella toxin can encode two or more UmbA proteins (Table S6, Figure 6A-C). Among these species, 77% encode more than one UmbC protein, indicative of secretion of multiple umbrella toxin particles. Remarkably, we identified eight species encoding ≥10 UmbA and ≥5 UmbC (Table S6). If we assume one cognate UmbA per UmbC as in *S. coelicolor*, these organisms could theoretically produce >10,000 morphologically and compositionally distinct umbrella particles. Interestingly, this analysis revealed a high degree of variability in the number of UmbA and UmbC proteins encoded at the strain level. For example, unlike the *S. griseus* strain we employed in this study (NBRC_13350), the majority of sequenced isolates of this species encode multiple UmbA proteins and many also encode multiple UmbC proteins (Table S6).

Using domain scanning, network clustering analyses and structural predictions, we further interrogated the total diversity of UmbA lectins within Streptomycetaceae. We found the C-terminal domains of *Streptomyces* UmbA proteins represent 41 distinct families, including 10 distinct protein folds (Figure 6D,E and Table S6). The two UmbA proteins for which we identified a target molecule, UmbA4^Sc^ and UmbA^Sg^, share the 6-bladed β-propeller fold, but belong to two distinct families (6BP-4 and 6BP-2, respectively), consistent with family level diversity among UmbA proteins operating as a minimal proxy for the extent of binding diversity. Given the sequence and protein fold diversity these families represent, we speculate that the surface carbohydrates exploited as receptors for umbrella toxins can vary widely. This is supported by our observation that UmbA5^Sc^, which possesses a 5-bladed β-propeller lectin domain, targets a bacterium that lacks a recognizable TUA biosynthetic gene cluster (Figure 1G and Figure 3C).

Additionally, we observe many instances in which individual UmbA proteins contain more than one lectin-like domain (13%, 1282 proteins), as observed for UmbA4^Sc^ (Table S6). Taken together with our genetic and structural analysis demonstrating a single lectin domain is sufficient to direct target cell recognition, this raises the possibility that UmbA proteins with multiple lectin domains could add further target range to individual umbrella particles. In total, our bioinformatic data demonstrate that encoding multiple UmbA and UmbC proteins is commonplace and strongly suggest that the two-tiered bet hedging strategy we observed in *S. coelicolor* constitutes a widespread evolutionary solution for interspecies antagonism permitted by the unique architecture of umbrella toxins (Figure 6F).

## Discussion

We present evidence that the modular, five spoked nature of umbrella toxin particles represents an unprecedented, bet hedging-driven adaptation to the unpredictable nature of competitive interactions between *Streptomyces*. Bet hedging as an evolutionary strategy in bacteria is typically associated with phenotypic variation across a population, wherein a subpopulation of isogenic clones express traits maladaptive for prevailing conditions, but which promote population survival during environmental fluctuations^14,15,32^. In contrast, by releasing combinatorial cocktails of umbrella toxins into the environment, *Streptomyces* bet hedge against the possibility of encounters with competitors in a manner that provides a benefit to the entire population. The cost of producing numerous individual toxin modules, only a subset of which will be active against a given competitor, is thus borne by the entire population.

Modularity is a common feature of polymorphic toxins^33,34^. However, this typically occurs within a single polypeptide and swapping is mediated by recombination. Umbrella toxins employ receptor binding and toxin domains encoded on separate polypeptides that interact via the adaptor protein UmbB (Figure 1A)^16^. This arrangement allows for efficient generation of particle compositional variation by a single strain. It also makes possible two tiers of bet hedging: the varying lectin domains present on UmbA enable recognition of different competitors, and the different toxin domains supplied by the UmbC proteins hedge against variable toxin susceptibility (Figure 6F). While we have observed examples of differential susceptibility to particular UmbC toxins across *Streptomyces* species, the molecular basis for this is not yet understood.

The structure we determined of UmbA4 in complex with TUA purified from *S. griseus* provides an unprecedented view of the contacts made between a β-propeller lectin and its natural ligand. This revealed just two of the six potential binding sites of UmbA4 make significant contact with TUA. While we cannot rule out that one or more of the four unoccupied sites might bind TUA in another context, none appear sterically occluded in our structure. Moreover, we found that disruption of either of the two sites we defined significantly reduces toxicity of the umbrella toxin particle toward *S. griseus*. These observations are consistent with the sequence divergence among UmbA4 blades and prior work suggesting that oligosaccharides approximating the physiological ligands of β-propellers are accommodated at fewer blade positions than are their simplified derivatives^31,35,36^. We speculate that limiting the binding of a particular TUA to a subset of β-propeller blade positions in single UmbA proteins could provide adaptive benefits. For instance, such an arrangement could broaden the species targeted by an umbrella particle by allowing a single β-propeller to recognize multiple ligands.

To our knowledge, the TUA–WTA polymer we found can serve as an umbrella toxin receptor has not been observed previously. While the structure of TUA polymers in *Streptomyces* has received some attention^22^, the biological function of these molecules remains obscure. We readily obtained mutants lacking TUA biosynthetic capacity with no apparent growth defects *in vitro,* and our genomic analyses indicate TUA is not produced by all *Streptomyces* species, suggesting TUA production is dispensable for basic physiology. We posit that as a scaffold particularly amenable to modification, TUA may enable escape from recognition by the many threats targeting surface-associated molecules, including phage. Additionally, TUA may provide other benefits in the environment that are typically attributed to anionic cell wall polymers. In *Bacillus subtilis*, TUA is a component of the Pho regulon and is proposed to serve a role in conserving phosphate by substituting for WTA^37^. Given the linkage between WTA and TUA that we uncovered, it appears unlikely that TUA serves merely as a WTA equivalent under phosphate limitation in *Streptomyces.* Instead, TUA–WTA polymers could provide WTA-linked benefits such serving as a physical barrier to passage of harmful molecules, mediating attachment, or anchoring surface-associated proteins^38–41^.

Umbrella toxin particles and their individual components hold promise for a number of biotechnological applications. The vast diversity of lectin domains within UmbA proteins could be harnessed in situations where interaction with specific carbohydrates is of interest. Examples include detection of bacterial pathogens in food products, diagnosis of cancers based on alterations to cancer cell surface glycans, and generation of cancer biotherapeutics with lectin-based targeting moieties^42^. Intact or engineered umbrella toxin particles hold potential for development as antimicrobials. As we demonstrate here, their modular nature can be exploited to generate chimeric particles with programmable species specificity. Although to-date the only targets we have identified are *Streptomyces*, *umb* gene clusters are found across Actinobacteria^16^. This raises the possibility that umbrella toxin particles capable of intoxicating medically relevant Actintobacteria such as *Mycobacterium tuberculosis* and *Corynebacterium diptheriae* may await discovery. Advancing to targeting organisms beyond those naturally susceptible to umbrella toxins will likely require a mechanistic understanding of the steps downstream of receptor binding, an important avenue of future investigation.

### Limitations of the study

Although our work supports a model in which a molecular arms race between umbrella toxin producers and targets drives diversification of umbrella toxin lectins and their receptors, respectively, this has not been established in a focused evolutionary study, and other biological systems that interact with these molecules may also contribute to their variation. Additionally, our proposed TUA biosynthetic pathway is based on structure modeling-guided functional predictions of proteins encoded within the *utr* locus and has not yet been experimentally validated; therefore, this remains speculative. Finally, while our cryo-EM data provide unambiguous evidence that *S. griseus* TUA interacts with UmbA4, we have not quantified the strength of this interaction, nor biochemically supported our contention that, more generally, the specificity of UmbA–TUA interactions dictates the target profile of umbrella toxins.

## STAR Methods

### EXPERIMENTAL MODEL AND STUDY PARTICIPANT DETAILS

#### Strains, media and growth conditions

Strains employed in this study are listed in the Key Resources Table and Table S1. *Escherichia coli* strain DH5α was used for plasmid maintenance and ET12567(pUZ8002) for interspecies conjugation. *E. coli* strains were grown in Lysogeny broth (LB) at 37 ℃ with shaking at 200 r.p.m or on LB agar plates (2%, w/v). Strains of *S. coelicolor* A3(2), *S. griseus* (NRRL B-2682), *S. antibioticus* (NRRL B-1701), and *S. achromogenes* (SANT-13) were used for umbrella toxin receptor studies; other *Streptomyces* strains listed in the Table S1 were used in screening for umbrella toxin susceptibility. Unless otherwise noted, *Streptomyces* strains were cultivated in R5, TSBY, or 2xYT liquid media in baffled flasks with 3 mm glass beads, shaken at 28 ℃ and 220 r.p.m., or on ISP2, ISP4 or SFM agar plates (2%, w/v). Media were supplemented as needed with antibiotics, including apramycin, kanamycin, chloramphenicol, and trimethoprim (all used at 50 mg/L).

### METHOD DETAILS

#### Plasmid construction

Primers and synthetic DNA fragments used in this study were obtained from Integrated DNA Technologies and are listed in Table S7. Plasmid constructs were designed using Geneious Prime, generated via Gibson assembly, and confirmed by sequencing. *S.* *coelicolor* genetic manipulation was conducted using pKGLP2a with the apramycin resistance gene (*aac*(3)*IV*), as previously described^16^. To enhance the efficiency of *S. griseus* and S. *antibioticus* mutant generation, the *codA(sm)* counterselection marker and its promoter, amplified from pLQ752^43^, were introduced to pKGLP2a to generate pKGLP2a-*codA(sm)*. CodA, a cytosine deaminase, allows for counterselection via the addition of 5-fluorocytosine, which it converts into highly toxic 5-fluorouracil^44^. Constructs for introducing deletions in chromosome of *Streptomyces* strains using pKGLP2a or pKGLP2a-*codA(sm)* were generated through Gibson assembly with 1.5–2 kb arms flanking the modification site. The integrative vector pSET152, which directs integration at the *attB* phage attachment site, was used for gene expression and complementation in *Streptomyces*^45^. For expression of UmbA4 in *S. coelicolor* strains, the gene was fused sequentially at the C-terminus with a 2xGGGGS linker, a 3xK tag and a 6xH tag and at N-terminus with the *S. coelicolor* SCO4296 promoter (198 bp) by PCR, then inserted to pSET152 by Gibson assembly. UmbA4 variants with mutations in key residues involved in TUA interaction were generated by PCR or DNA synthesis. For *utrC* complementation in *S. griseus* Δ*utrC*, the gene was fused with the SCO4296 promoter at and cloned into pSET152.

#### Genetic manipulation of *Streptomyces* strains

To generate genetically modified *S. coelicolor*, vectors derived from pKGLP2a with the corresponding inserts were introduced via *E. coli*-*Streptomyces* intergeneric conjugation using *E. coli* ET12567(pUZ8002) as the donor strain. Conjugation and mutant screening were performed as described previously^16^. For gene deletion in *S. griseus* and *S. antibioticus*, derivatives of pKGLP2a-*codA(sm)* carrying homologous alleles were transferred via the same conjugation method as for *S. coelicolor*, with an extended 50℃ heat-shock treatment of spores (20 min) before mixing with donor *E. coli* cells. Transconjugants were streaked onto ISP4 agar supplemented with trimethoprim and apramycin and then cultured in non-selective TSBY medium for 24 h. The cultures were subsequently streaked on non-selective ISP4 agar and incubated at 30 ℃ for 5–7 days to promote sporulation. Spores were collected, diluted, and plated on ISP4 agar containing 50 mg/L 5-fluorocytosine. After 2 days of incubation, colonies were screened for apramycin sensitivity and the presence of the desired allele via PCR. For gene expression or complementation in *Streptomyces* strains, the integrative vector pSET152 carrying the desired genes and corresponding promoters was introduced through conjugation, following the procedure species-specific procedures described above for pKGLP2a and pKGLP2a-*codA(sm)*.

#### Preparation of concentrated supernatant from *Streptomyces* strains

Cultures of *S. coelicolor* used for concentrated supernatant preparation were grown as previously described^16^. For preparation of *S. griseus* concentrated supernatant (sup^Sg^), spores were grown in 30 mL R5 medium for 24 h, then back diluted to an OD_600_ of 0.005 in 50 mL R5 medium per flask, with a total combined volume of 150 mL. Back-diluted cultures were incubated for approximate16 h until OD_600_ reached 4–5. For both species, cultures were centrifuged to remove cells, resulting supernatant was filtered with a 0.45 μm PES membrane vacuum filter and then concentrated using 100 kDa cutoff concentrators (Amicon) until reaching a final volume of 3 mL. Concentrated supernatants were desalted using Econo-Pac 10DG desalting columns (Bio-Rad), filter sterilized (0.22 μm), aliquoted and stored at −80 ℃.

### Isolation and identification of *Streptomyces* strains from soil and root samples

We grew *Arabidopsis thaliana* (Col-0) under standardized conditions in natural soils from diverse U.S. regions. After 8 weeks, we collected bulk soil and roots for bacterial culturing. To enrich for endophytes, roots were vortexed and sonicated in sterile water, then homogenized and serially diluted (1/10) for plating on ISP4 and water agar; bulk soil was treated similarly. *Streptomyces*-like colonies were re-streaked twice on ISP4 to obtain pure cultures. Spores were collected in 40% glycerol and stored at −80 °C. To identify isolated *Streptomyces* species, genomic DNA was extracted from 20 h cultures using InstaGene Matrix (Bio-Rad), and the V1-V3 region of the 16S ribosomal RNA gene was amplified by PCR and analyzed by Sanger sequencing (Azenta). Species identification was performed using a BLAST search of these sequences against the 16S ribosomal RNA sequences (Bacteria and Archaea) database.

#### Screening *Streptomyces* strains for sensitivity to Umb toxins

To screen for new target strains sensitive to *S. coelicolor* Umb particles, 19 diverse *Streptomyces* species (see Table S1 for complete list) were streaked on ISP4 and grown for 24–72 h before being inoculated into 100 μL TSBY cultures in a 96-well plate. The plate was incubated at 30℃ for 36 h in a BioTek LogPhase 600 Microbiology Reader with shaking at 800 r.p.m. Optical densities of the initial cultures were measured and diluted to an OD_600_ of 0.001 in TSBY medium. Next, 90 μL aliquots of each test strain culture were mixed with 10 μL of sup^Sc^ derived from either wild-type or Δ*umbC1–3* (negative control) in a 96-well plate. The plate was incubated at 30℃ for 16 h. Cell viability of the cultures was assessed by combining each with 100 μl BacTiter-Glo reagent (Promega), incubating for 5 min at room temperature, and measuring luminescence using a BioTek Cytation 1 imaging reader. To identify a strain sensitive to the *S. griseus* Umb particle, 37 diverse *Streptomyces* species (listed in Table S1) were screened using the same procedure described above, with minor modifications. In brief, 99 μL diluted culture aliquots were mixed with 1 μL sup^Sg^ from either wild-type or Δ*umbC S. griseus* (negative control). Cell viability was determined using the BacTiter-Glo reagent after incubation for 16 h. For both screens, growth inhibition was determined by calculating the ratio of luminescence signals from cultures treated with Δ*umbC* supernatant to those treated with wild-type supernatant. Two biological replicates were performed for each screen, and Z scores were calculated from the average of log_2_-transformed ratios.

#### Evaluation of Umb particle toxicity

Methods of assessing the toxicity of Umb particles using concentrated supernatants from *S. coelicolor* wild-type and mutant strains (sup^Sc^) and *Streptomyces* co-culture competition assays have been described previously^16^. To evaluate the toxicity of Umb particles produced by *S. griseus,* concentrated supernatants from *S. griseus* wild-type and mutant strains (sup^Sg^ ) were prepared as described above. *S. antibioticus* precultures (grown for 20 h, as described above) were diluted to an OD_600_ of 0.01 in TSBY medium. A 99-µL aliquot of the diluted culture was mixed with 1 µL of sup^Sg^ in a 96-well plate and then incubated in a LogPhase for 16 h. Growth of *S. antibioticus* was quantified by OD_600_ measurement after 16 h of incubation.

#### Bacterial pull-down assay and whole cell proteome analysis

For detection of proteins that bind to Umb target cells, spores of target organisms *S. griseus* and *S. antibioticus* were cultured in 30 mL TSBY medium for 20 h. Cells were pelleted by spinning at 3,000 g for 5 min, washed twice with TSBY medium, and resuspended in TSBY medium to an OD_600_ of approximately 4. A 500 μL cell aliquot was mixed with 500 μL of sup^Sc^ (from *S. coelicolor* Δ*umbC1–3*), sup^Sg^ (from *S. griseus* Δ*umbC*), a 1:1 (v/v) mixture of sup^Sc^ and sup^Sg^, or PBS (negative control) in a 1.5 mL microcentrifuge tube. Samples were incubated at RT with continuous rotation for 1.5 h. Then cells were pelleted by spinning at 3,000 g for 5 min, washed three times with PBS, resuspended in 400 uL lysis buffer (8 M urea, 75 mM NaCl, 50 mM Tris-HCl, pH 8.2) and lysed by sonication. After spinning at 20,000 g for 20 min, 20 μL of resulting supernatant was used to determine protein concentration via the Pierce™ BCA Protein Assay Kit (Thermo Fisher Scientific). Meanwhile, 100 μL of protein solutions were transferred to a new 1.5 mL microcentrifuge tube and incubated with 5 mM Dithiothreitol (DTT) at 56 ℃ for 25 min for protein reduction. After cooling to room temperature, 14 mM iodoacetamide was added for protein alkylation and the samples were incubated in the dark at room temperature for 30 min. The reaction was quenched by adding additional 5 mM DTT and incubating 15 min in the dark at room temperature. The mixture was diluted 5-fold with 50 mM Tris-HCl (pH 8.2) to reduce the urea concentration to 1.6 M and 1 mM calcium chloride was then added. Approximately 20 μg protein (as determined by BCA assay) was mixed with trypsin (Promega) at 4 μg/mL and incubated at 37 ℃ for 16 h. Digestion was halted by adding 0.4% trifluoroacetic acid (TFA) to lower the pH below 2.

Trypsin-digested peptides were purified using in-house constructed stop-and-go-extraction tips (StageTips)^46^ embedded with 6 layers of Empore™ styrene divinyl benzene (SDB-RPS) extraction disks (Sigma). StageTips were conditioned sequentially with 50 μL of 100% methanol, 100% acetonitrile (ACN), 75%ACN and 5% ammonium hydroxide, 75% ACN and 0.5% acetic acid, and 0.1% TFA. Digested peptide samples were loaded onto StageTips and washed sequentially with 50 μL of 0.1% TFA, 75% ACN and 0.5% acetic acid, 0.5% acetic acid, then eluted with 60 μL of 75%ACN and 5% ammonium hydroxide. Peptides were dried using a SpeedVac™ vacuum concentrator (Thermo Fisher Scientific) and resuspended in 5% ACN with 0.1% formic acid and analyzed by LC-MS/MS on a Lumos Fusion Orbitrap mass spectrometer (Thermo Scientific) as previously described^47^.

For analysis of proteins in sup^Sc^ and sup^Sg^ (“reference”), 100 μL of protein solution was mixed with 25 μL 100% trichloroacetic acid (TCA) and incubated on ice for 30 min to precipitate proteins. After centrifugation at 20,000 x g for 5 min, the protein pellet was washed once with 20% TCA and twice with 100% acetone. The dried protein samples were resuspended in lysis buffer (8 M urea, 75 mM NaCl, 50 mM Tris-HCl, pH 8.2) and subsequent reduction, alkylation, trypsin digestion, and peptide purification and mass spectrometry were performed as described above.

Mass spectrometry data were analyzed using MaxQuant^48^. Data were filtered to remove proteins identified in the negative control, where cells were incubated with PBS instead of supernatant. The proportion of mass spectral counts for proteins in “reference” and “cell-associated” groups was then calculated. Two biological replicates were included in each group.

#### Adaptive laboratory evolution of Umb-resistant strains

To establish clonal ancestor populations for these experiments, wild-type *S. griseus* and *S. antibioticus* strains were streaked onto ISP4 agar and incubated at 30 ℃ for 5–7 days until isolated colonies sporulated. Spores from a single colony were collected in 20% glycerol (w/v) using a sterilized cotton swab, streaked onto multiple ISP4 agar plates, and incubated until sporulation. The spores were then collected in 20% glycerol (w/v), aliquoted, and stored at −80℃.

To determine the minimum inhibitory concentration (MIC) of sup^Sc^ toward *S. griseus*, sup^Sc^ was serially diluted 2-fold in sup^Sc^ from *S. coelicolor* Δ*umbC2* to create working solutions with different concentrations of Umb2 particles. *S. griseus* clonal spores were cultured in 30 mL TSBY medium for 20 h, then back diluted to an OD_600_ of 0.01. A 90 μL aliquot of the diluted culture was mixed with 10 μL of each working solution in a 96-well plate. The plate was incubated in a LogPhase at 30 ℃ for 16 h, and cell viability was assessed using BacTiter-Glo reagent. Data were normalized to the range of growth observed in positive (undiluted wild-type sup^Sc^) and negative (Δ*umbC2* sup^Sc^) control treatments. A similar procedure was used to determine the MIC of sup^Sg^ against *S. antibioticus*, except cell growth was assessed by OD_600_ measurement. The concentrations of sup^Sc^ and sup^Sg^ that resulted in approximately 90% growth inhibition were selected as the initial treatment for evolution of Umb-resistant mutants (1.25% for sup^Sc^ and 0.12% for sup^Sg^).

For ALE of *S. griseus* mutants resistant to *S. coelicolor* Umb2 particles, *S. griseus* clonal spores were grown in TSBY medium for 20 h then diluted to an OD_600_ of 0.01. 5 mL aliquots of diluted culture were added to 100 mL baffled flasks containing 3 mm glass beads and mixed with sup^Sc^ from wild-type or *S. coelicolor* Δ*umbC2* mutant to a final concentration of 1.25% (v/v) (passage 1). Three independent lineages were treated with wild-type sup^Sc^, while one lineage treated with *S. coelicolor* Δ*umbC2* sup^Sc^ served as a negative control. After 48 h of incubation, cultures reached an OD_600_ of 8–9 and were reinoculated into fresh TSBY medium containing 1.25% sup^Sc^ to an OD_600_ of 0.01 to initiate passage 2. Meanwhile, 50 µL cells from passage 1 were streaked on ISP4 agar plates for sporulation. The liquid cultures reached OD_600_ of 2–7 after 24 h of incubation, and were streaked out again and rediluted to OD_600_ of 0.01 in TSBY medium containing 1.25% sup^Sc^ for passage 3. In this passage, the OD_600_ reached 8–10 within 24 h of incubation, and cultures were streaked for sporulation at this point.

Spores from different lineages and passages were collected, diluted, and plated on ISP4 agar for single colony isolation. Colonies were selected for sup^Sc^ sensitivity testing at random in equal numbers from each lineage for a total of 432 colonies from passage 1, 48 colonies from passage 2, and 24 colonies from passage 3. Along with the ancestor strain, these individual colonies were used to inoculate 100 μL TSBY cultures in 96-well plates and incubated for 16 h at 30℃ in a BioTek LogPhase 600 Microbiology Reader. Optical densities of starter cultures were measured and used to inoculate 50 μL TSBY with 10% wild-type sup^Sc^ to an OD_600_ of 0.003 in new 96-well plates. These plates were incubated in a LogPhase for 16 h and growth was assessed by combining each with 50 μL BacTiter-Glo reagent and measuring luminescence after a 5-min incubation at room temperature.

*S. antibioticus* mutants resistant to *S. griseus* Umb particles were generated using a similar ALE procedure as described above, with the modifications noted below. In brief, pre-cultured *S. antibioticus* cells were diluted to an OD_600_ of 0.01 and treated with 0.12% (v/v) sup^Sg^ in passage 1. After 68 h of incubation, cultures reached an OD_600_ of 1.3–5.2 and were reinoculated into fresh TSBY medium containing 0.12% sup^Sg^ at an OD_600_ of 0.01, initiating passage 2. For subsequent passages, the MIC of each evolving lineage was determined as described above, and sup^Sg^ concentration used for selection was adjusted to maintain the new MIC, which reached 1% at passage 4. The ALE process was halted after passage 5, when the evolved populations had become resistant to >10% sup^Sg^. Isolation of single colonies from all lineages and passages was performed as described above, and 48 colonies each from passages 3, 4, and 5 were screened for sensitivity to 1% wild-type sup^Sg^.

#### Whole genome sequencing and sequence analysis

No reference genome sequence was publicly available for *S. antibioticus* NRRL B-1701. To obtain a reference genome for this species, an overnight culture of the strain was centrifuged to obtain a 30–50 mg cell pellet. This washed with 1 mL PBS, suspended in 0.5 mL Zymo 1X DNA/RNA Shield, and submitted to Plasmidsaurus for genome sequencing and annotation. To obtain sequences for identifying mutations in Umb-evolved strains, overnight cultures of evolved clones and of the relevant ancestral strains were grown and used for total gDNA extraction with the DNeasy Blood & Tissue kit (Qiagen). Sequencing libraries were constructed using the Nextera DNA Flex Library Prep Kit (Illumina) and sequencing was performed with an Illumina MiniSeq or an Illumina iSeq (300 cycles paired end program on each instrument). Reads were aligned to the *Streptomyces griseus subsp. griseus* NBRC 13350 (genome accession GCA_000010605.1) or *S. antibioticus* NRRL B-1701 reference genome using Geneious. Reads were trimmed using the BBDuk plugin. SNPs were called using Geneious with a minimum coverage cutoff of 5 and a minimum variant frequency of 0.9. SNPs present in the ancestor strain were filtered out of the results for the evolved isolates. Only SNPs present in coding sequences were considered. Sequencing data for evolved isolates and the genome sequence of *S. antibioticus* NRRL B-1701 are available under BioProject PRJNA1254743.

#### Wall teichoic and teichuronic acid characterization

##### Isolation of wall teichoic and teichuronic acids

Isolation of wall teichoic and teichuronic acids (WTA and TUA) from *Streptomyces* cells was performed essentially as described previously^21^. In a typical preparation, 4 g of cells in a 50 mL conical centrifuge tube were suspended in 10 mL of cold 2 M NaCl by pipetting, 25 g 0.1 mm zirconia/glass beads (BioSpec Products) were added and the tube was cooled to 4 °C. The sample was vortexed in the cold room four times for 2 min at highest speed (Vortex Genie 2), with breaks between the cycles to allow for cooling of the material. Samples were then centrifuged for 1 min at 1000×*g*, the cell debris suspension was removed, and the beads were washed twice using 10 mL 2 M NaCl, with centrifugation. The combined cell suspension was centrifuged for 15 min at 14,000×*g*, the supernatant was discarded, the pellet resuspended in 20 mL of phosphate-buffered saline (137 mM NaCl, 27 mM KCl, 10 mM Na_2_HPO_4_, 1.8 mM KH_2_PO_4_) and centrifuged again. The pellet was washed in a similar manner by resuspension (20 mL each) and centrifugation three times with 2% sodium dodecylsulfate and three times with ultrapure water. The remaining material, resuspended in 20 mL H_2_O, was incubated at 60 °C with shaking, pelleted and resuspended in 20 mL of 15 mM Tris, pH 7.0 buffer with 4 mg trypsin. Following an overnight incubation at 37 °C with shaking, the sample was centrifuged and the pellet washed with (20 mL each) 1 M Tris, pH 7.0, 1 M Tris with 1 M NaCl and 1 M Tris, followed by three washes with ultrapure water. The resulting pellet was suspended in 10 mL cold 10% trichloroacetic acid (TCA) and rocked overnight at 4 °C. The sample was then centrifuged 45 min at 10,000×*g* at 10 °C, the supernatant removed, combined with 1/10 volume of 3 M sodium acetate, pH 5.1, and chilled at 4 °C. To the solution, three volumes of cold 95% ethanol was added and the mixture was incubated at −18 °C for at least 16 h. The sample was then centrifuged 40 min at 10,000×*g* at room temperature, the supernatant carefully removed and the pellet of released wall teichoic acid washed from the tube wall with 2 mL 95% ethanol twice. The suspension was centrifuged 5 min at 16,000×*g* and the pellet washed by centrifugation five times with 95% ethanol (1.5 mL each). The residual ethanol was dried on a centrifugal vacuum evaporator, the pellet dissolved in 0.6 mL ultrapure water and centrifuged 5 min at 16,000×*g* to remove water-insoluble material. The solution was lyophilized and the wall teichoic acids were analyzed as described below.

##### Dephosphorylation

Lyophilized wild-type *S. griseus* TUA–WTA (1.4 mg) was dissolved in 300 μL cold 48% hydrofluoric acid (HF) on ice and the solution was kept at 4 °C for 30 h. HF was evaporated with a stream of air to ~30 μL, diluted with ultrapure water to 300 μL and evaporated again. Following dilution to 300 μL, the sample was lyophilized and fractionated using size exclusion chromatography (SEC).

##### Size exclusion chromatography (SEC)

SEC was performed on a Superdex 75 10/300 GL column (Cytiva) connected to an Agilent 1260 Infinity II high-performance liquid chromatography system equipped with a refractive index detector (RID) for monitoring eluting species. The separations of the intact (50 μg) and dephosphorylated (1.4 mg) TUA–WTA material isolated from wild-type *S. griseus* (dissolved in water) were performed at a flow rate of 0.5 mL/min of 50 mM ammonium acetate buffer (pH 6). A semi-preparative SEC of the wild-type *S. griseus* TUA/WTA dissolved in the running buffer (two runs of 1.3 mg each) was performed in 100 mM ammonium acetate (pH 6). Because of the shift between RID and fraction collector, each fraction was analyzed by ^1^H NMR and assessed for the presence of signals characteristic for TUA and WTA. Representative early and late fractions were also analyzed by ^1^H,^13^C-HSQC and DOSY. Based on the results, fractions within 10–12 ml elution volumes were pooled as those containing TUA+WTA, and the WTA-only containing fractions from elution volumes 13–15 ml were pooled separately. The structures present in each pool were determined by 1D and 2D NMR, as described below.

##### Mass spectrometry analysis

Mass spectrometry (MS) analysis of the dephosphorylated *S. griseus* TUA material was performed by the matrix-assisted laser desorption/ionization time-of-flight (MALDI-TOF) technique on a Bruker rapifleX mass spectrometer. Mass spectra of the TUA mixed with SDHB matrix (9:1 2,5-dihydroxybenzoic acid:2-hydroxy-5-methoxybenzoic acid) were acquired in the reflector negative mode. The spectrometer was calibrated using angiotensin-I peptide and the spectra were analyzed in Bruker Daltonik FlexControl 3.0 software.

##### NMR spectroscopy

Each NMR sample was prepared from 1–2 mg of dry material that was first dissolved in 200 μL D_2_O (99.9% D, Sigma) with 60 nmol DSS-*d*_6_ (Cambridge Isotope Laboratories) and lyophilized. Following the deuterium exchange, the sample was dissolved in 42 μL D2O (99.96% D, Thermo Scientific) and transferred into a 1.7 mm NMR tube. For *S. griseus,* a sample of the SEC-separated higher mass material was also prepared in 97:3 H_2_O:D_2_O solvent to observe signals due to amide hydrogens. NMR data were acquired on a Bruker Avance NEO NMR spectrometer (^1^H, 799.71 MHz) equipped with a 1.7 mm TCI cryoprobe. The pulse programs included in the spectrometer library were used to acquire 1D ^1^H and 2D COSY, TOCSY, NOESY, ROESY, DOSY, ^1^H,^13^C-HSQC, ^1^H,^13^C-HMBC and ^1^H,^13^C-HSQC-TOCSY data. A ^13^C-coupled IPAP-HSQC experiment was acquired to determine ^1^H–^13^C ^1^*J* scalar couplings and confirm the anomeric α/β configurations. Quantitative ^1^H NMR data were acquired with a total recovery delay of 60 s. Mixing time was set to 80 ms for NOESY and 200 ms for ROESY experiments, to 70 and 140 ms for TOCSY experiments, and to 70 ms for HSQC-TOCSY experiment. The DOSY experiments were run using a stimulated echo with longitudinal eddy current delay and bipolar gradient pulses. Gradient strength was varied from 3 to 97% maximum power in 32 linear increments. The diffusion gradient length was 2 ms and diffusion delay (400–600 ms) was optimized for each sample. The NMR data for *S. griseus* samples were acquired at 313 K, except for the DOSY spectra acquired at 298 K. The NMR data for *S. antibioticus* samples were acquired at 298 K. ^1^H and ^13^C chemical shifts were referenced to the respective DSS signals at 0.00 ppm. NMR data were processed in Topspin 4.0.5, analyzed in AnalysisAssign 3.2.0^49^ and rendered for figures in MestReNova (14.2.3). DOSY reconstruction was done in MestReNova using the peak fit algorithm.

##### Structural analysis of S. griseus TUA–WTA

NMR spectra of TCA-released material from the wild-type *S. griseus* cell walls indicated that it contained six distinct glycosyl residues that were labeled A–G (Table S4, Figure S2A). The highest-abundance residues C and E were identified as hexopyranoses bearing acetamido groups. Residue C was found to be a 3-substituted α-GalNAc based on the characteristic chemical shifts and the interruption of ^1^H–^1^H spin system at H-4. Residue E was a 2,3-diacetamido-2,3-dideoxyhexuronic acid in *manno* configuration, given the break of the ^1^H–^1^H spin system at H-2 and an HMBC correlation from H-5 to a carbonyl resonance at 176.6 ppm that shifted to lower field at higher pH. The β anomeric configuration of residue E was confirmed by the anomeric ^1^*J*_HC_ of 163 Hz and ROESY interactions H-1–H-3 and H-1–H-5. A disaccharide repeating unit consisting of residues C and E was established based on the glycosidic linkage signals in the NOESY/ROESY and HMBC spectra (Figure S2A) to be →3)GalNAc-α(1→4)-ManNAc3NAcA-β(1→. Chemical shifts of this disaccharide motif agreed closely with those previously reported for TUAs isolated from *S. lavendulocolor* and *S. griseus*^22,50^. From the chemical shift agreement, we inferred that both residues were in the D absolute configuration as reported.

In addition to the major residues C and E, weaker signals were present that belonged to four residues constituting the terminal and linker groups. We found that two non-reducing end residues were present, suggesting irregular termination of the chain. Majority of the polymer (~70%) was terminated by 4-*O*-acetylated β-ManNAc3NAcA (residue D), while ~30% of the chains were terminated by α-GalNAc (residue B). According to the HMBC and ROESY correlations (Figure S2A), residue D formed a β(1→3) glycosidic bond with a C-like residue, while residue B was α(1→4) linked to an E-like residue, consistent with an alternate termination of the chain.

On the reducing end of the chain, two distinct glycosyl residues were present, a 3-substituted Gal*p*NAc (residue A) that was α(1→4) linked to a Glc*p*NAc3NAcA (residue G). Residue G was identified as a 2,3-diacetamido-2,3-dideoxyhexuronic acid in *gluco* configuration owing to an uninterrupted ^1^H–^1^H spin system from H-1 to H-5 and an HMBC correlation from H-5 to a carbonyl resonance at 175.2 ppm that was pH-dependent. The β anomeric configuration of residue G was confirmed by the anomeric ^1^*J*_HC_ of 164 Hz, ^3^*J*_HH_ between H-1 and H-2 of 7.8 Hz, and characteristic ROESY interactions. ^13^C chemical shifts of this A–G disaccharide matched well those reported for a TUA with a →3)-D-GalNAc-α(1→4)-D-GlcNAc3NAcA-β(1→ repeat that was found in *Streptomyces* sp. VKM Ac-2537^22^; the matching chemical shifts indicate the D absolute configuration of both residues A and G. Based on a ROESY correlation (Figure S2A), the 3-OH of residue A was substituted by an E-like residue. The terminal residue G was then glycosidically linked by a β(1→4) bond to a ribitol-5-phosphate residue (Rg), as apparent from the ROESY and HMBC spectra (Figure S2A).

NMR analysis of the larger-size TUA–WTA molecule obtained by SEC as well as the dephosphorylated TUA both corroborated the structure of the TUA-like glycan and its linkage to a ribitol-5*P* residue (Figure S2B-D, Table S4). In addition to the major glycosyl residues, a small amount of terminal ManNAc3NAcA was identified (residue K) that was likely the product of limited de-O-acetylation of residue D. A partially resolved residue (Cd) was also identified in the SEC-separated TUA–WTA species that was the aglycon of the non-reducing end residue D. NMR data acquired in 97:3 H_2_O:D_2_O provided spectral information for the amide HN groups (Table S4) and were instrumental in verifying the chemical shift assignments and connectivities for the mono- and diamino-hexoses.

In the dephosphorylated TUA, NMR showed that the reducing-end residue G was β(1→4) linked to a terminal ribitol (residue Rh). MALDI-TOF MS analysis of the dephosphorylated TUA (Figure 2H) was consistent with the TUA structure determined by NMR. Two series of peaks were present in the MALDI spectrum that corresponded to the two polymers with alternative non-reducing ends and with varying numbers of the disaccharide repeating unit (*m/z* differences of 461.16). Masses corresponding to 2–6 repeats with the 4OAc-ManNAc3NAcA terminus, as well as the de-*O*-acetylated equivalents were present. Similarly, masses corresponding to 1–6 repeats with the GalNAc terminus were detected.

##### Structural analysis of S. griseus WTA

Analysis of polyol phosphates present in the WTA portion of the TCA-released material from the cell walls of wild-type *S. griseus* was based mainly on the HSQC, HSQC-TOCSY and HMBC spectra that circumvented the overlaps present in the ^1^H–^1^H correlation spectra. The analysis provided individual polyol*P* units but their connectivities could not be defined using the above experiments. ^1^H,^31^P-HMBC spectrum was also acquired in an attempt to establish sequential connections of the polyols through phosphodiester groups, but signal crowding prevented its meaningful analysis.

Based on the HSQC-TOCSY and HMBC correlations as well as characteristic ^1^H/^13^C chemical shifts^26,27,51^ and symmetry considerations, three major polyol*P* groups were identified in the total TCA hydrolysate as well as the SEC-separated TUA–WTA and WTA material (Table S4): a 1,5-linked Rbo*P* (residue Ra), a 1,3,5-linked Rbo*P*_*2*_ (residue Rb) and a terminal 3-linked Gro*P* (residue Gr). Low-abundance terminal 3-linked Rbo*P* (residue Re) was also found in the lower-mass WTA material (Table S4). The composition of the polyol*P* residues indicated that *S. griseus* WTA is mainly the poly(Rbo*P*) type and may contain Gro*P* branches or termini. A very low level of lysine modification was also detected in all WTA preparations.

Analysis of the *S. griseus* ∆*utrC* WTA material showed that it predominantly consisted of 1,5-linked Rbo*P* (Ra) with reduced amounts of terminal Gro*P* (Gr) residue (Table S4). The mutant WTA contained a terminal Rbo-5*P* (residue Rc) that was not observed in the wild-type; however, residue Rc could be analogous to the wild-type residue Rg that is not substituted with the TUA. The content of branching Rbo*P*_*2*_ (Rb) was very low in the mutant WTA. An additional, unidentified polyol*P* (residue Rd) was also present.

##### Structural analysis of S. antibioticus TUA-WTA and WTA

Based on the analysis of NMR spectra, the TCA-released material from wild-type *S. antibioticus* contained seven distinct glycosyl residues, labeled A–G (Table S4, Figure S4). A subset of the residues (C, D, F, G) was each found to be a 2,3-diacetamido-2,3-dideoxyhexuronic acid in *gluco* configuration owing to an uninterrupted ^1^H–^1^H spin system from H-1 to H-5 and an HMBC correlation from H-5 to a carbonyl resonance at ~173 ppm (low pH). The most abundant residue C formed a β(1→4) linked homo-oligomer, →4)-Glc*p*NAc3NAcA-β(1→, as was apparent from the HMBC and ROESY inter-residue correlations. Residue F was the non-reducing end of the oligomer bearing a 4-O-acetyl group. On the reducing end, a partially resolved GlcNAc3NAcA (residue D) connected the chain to a terminal Glc*p*NAc3NAcA with distinct chemical shifts (residue G); residue G then linked the oligomer to a Rbo-5P residue (Rg) via a β(1→4) glycosidic bond. The absolute configuration of the GlcNAc3NAcA residues has not been determined. To the best of our knowledge, a homopolymer of GlcNAc3NAcA has not been reported previously.

The remaining three residues (A, B, E) were identified as terminal β-Glc*p* residues glycosidically linked to the polyol*P* backbone of the WTA (Figure S4A,D). The most abundant residue A connected via a β(1→4) bond to 1,5-linked ribitol-5P unit (residue Rf). A small proportion of the Glc residues were 3-O-methylated (residue B). A similar glucosylated Rbo-5*P* polymer has been reported in WTA from other *Streptomyces*^26,50^. The WTA also contained unsubstituted 1,5-linked ribitol-5*P* residues (Ra). A minor residue E was then β(1→4) glycosidically linked to another polyol*P* that has not been identified.

The spectra of *S. antibioticus* ∆*utrC* TCA-released material lacked signals of the TUA-like residues C, D, F and G, and contained only signals of glycosyl residues A, B and E, together with ribitol-*P* residues Ra and Rf (Table S4, Figure S4A,B). Additionally, similar to what was observed for *S. griseus* ∆*utrC*, a terminal Rbo-5*P* was present (residue Rc) that could be analogous to the wild-type residue Rg not substituted with the TUA-like chain in the mutant.

#### Purification of UmbA4–UmbB

*S. coelicolor* Δ*umbC1–3* pSET152–*attB*::*umbA4*–2xGGGGS–3xK–6xH was used for purification of UmbA4–UmbB complexes. Spores were cultured in 30 mL 2xYT broth (Research Products International) for 24 h, then back diluted to an OD_600_ of 0.01 in 100 mL 2xYT broth for a total combined culture volume of 4 L. Cultures were incubated for approximately 28 h until OD_600_ reached 5–6. Cells were pelleted by centrifugation at 21,000 g for 30 min, and the supernatant was filtered using Rapid-Flow™ Filter Units (PES Membrane, 0.45 um; Thermo Fisher Scientific). A total of 3.5 L of supernatant was combined with 700 mL of 5xPBS buffer and incubated with approximately 10 mL bed volume TALON cobalt resin (Takara) under continuous stirring at 4 ℃ for 4 h. The resin was separated from supernatant by centrifugation at 300 g for 3 min and packed into two Econo-Column^®^ Chromatography Columns (2.5 × 30 cm; Bio-Rad). The approximately 4 L supernatant was loaded onto the columns, followed by washing with PBS buffer. Bound proteins were eluted with PBS containing 500 mM imidazole. Eluted fractions were concentrated using a 30 kDa cut-off Amicon concentrator to a final volume of 1.1 mL. The protein sample was further purified by AKATA FPLC using a Superose 6 Increase 10/300 GL column (GE healthcare) equilibrated in sizing buffer (250 mM NaCl, 2.7 mM KCl, 8 mM Na_2_HPO_4_, and 2 mM KH_2_PO_4_, pH 7.2). Each fraction was assessed for purity by SDS-PAGE with Coomassie blue staining. Fractions with high purity and concentration were further concentrated and used for Cryo-EM or cell binding assays.

#### Fluorophore-conjugated UmbA4 binding assays

For conjugation with the fluorophore Tetramethylrhodamine (TRITC), purified UmbA4 protein with a 3xK tag at the C-terminus (0.3 mg/mL) was mixed with 100 mM sodium bicarbonate buffer (pH 8.3) to adjust the reaction pH. TRITC, dissolved in DMSO, was then added to achieve a 1:10 molar ratio (UmbA4 : TRITC). The mixture was incubated at room temperature for 1.5 h with continuous agitation at 2,000 r.p.m in a Thermal Mixer (Thermo Fisher Scientific). The reaction was quenched by adding 100 mM NH_4_Cl (pH8.3), followed by a 1-h incubation at room temperature. Unconjugated TRITC and other molecules were removed via desalting using a PD SpinTrap G-25 column (Cytiva) equilibrated by PBS buffer. The labeled UmbA4 protein was aliquoted and stored at −80 ℃.

For the binding assay, *S. griseus* spores were grown in TSBY medium for 16 h. Cultures were pelleted by spinning at 3000 g for 5 min, washed once with LB broth, and resuspended in LB to an OD_600_ of 20. A 7-µL cell suspension were mixed with 7 µL of labeled UmbA4 protein or PBS (negative control) and incubated at 1,000 r.p.m in a Thermal Mixer for 40 min. Cells were then pelleted (3000 g, 5 min), washed twice with 500 µL of a solution containing 50% LB and 50% PBS, resuspended in 50 µL of the same solution, and transferred into a 96-well plate (Thermo Scientific^™^ Nunc MicroWell 96-Well Optical-Bottom Plates). Fluorescence intensity was measured using a CLARIOstar Plus plate reader (BMG Labtech) with excitation at 539/15 nm and emission at 582/20 nm. Background fluorescence from PBS-incubated controls was subtracted.

To quantify cells used in the binding assay, 50 µL of the suspension used for fluorescence measurement was mixed with 50 µL of BacTiter-Glo reagent (Promega) in a white 96-well opaque plate, and luminescence was measured after a 5-min incubation at room temperature. Fluorescence intensity was normalized to cell number and then scaled to the maximum and minimum values within the assay. Three biological replicates were included.

#### Cryo-EM-based structural studies

##### Sample preparation, data collection and processing

Structural studies of UmbA4–UmbB complexes in the absence of bound TUA were carried out by mixing three μl of 3 mg/mL purified protein complex with 2 mM 3-[(3-Cholamidopropyl)dimethylammonio]-2-hydroxy-1-propanesulfonate (CHAPSO) before loading onto freshly glow discharged R 2/2 UltrAuFoil grids, prior to plunge freezing using a vitrobot MarkIV (ThermoFisher Scientific) with a blot force of 0 and 5 sec blot time at 100 % humidity and 22°C. Cryo-EM analysis revealed that each purified fraction contained various levels of UmbA4 alone and UmbA4 complexed with UmbB (co-purified UmbB1–3 could not be distinguished). To obtain a structure of UmbA4–UmbB, 2,380 micrographs were collected using a TFS Glacios operated at an accelerating voltage of 200 kV with Gatan K3 direct electron detector, a nominal magnification 45,000x, a calibrated pixel size of 0.89 Å, an exposure time of 5 s and a total fluence of 47.3 e/Å^2^, using the Leginon software^52^. Movie frame alignment, estimation of the microscope contrast-transfer function parameters, particle picking and extraction were carried out using in cryoSPARC^53^. 2D classification, ab-initio reconstruction, heterogeneous 3D refinement and non-uniformed 3D refinement was performed in cryoSPARC ^54^. The final 3D refinement of UmbA4 protein was carried out in cryoSPARC, leading to a map with resolution of 4.3 Å comprising 200,137 particles.

To determine the structure of UmbA4 bound to TUA, 50 µM protein and 1 mM teichuronic acid (TUA), purified via dephosphorylation and SEC from *S. griseus* TUA–WTA, were incubated in buffer containing 10 mM Na_2_HPO_4_, 1.8 mM KH_2_PO_4_ pH 7.4, 250 mM NaCl and 2.7 mM KCl for 60 min at 4 °C. Subsequently, three microliters of 3.5 mg/mL UmbA4–TUA with 2 mM CHAPSO were loaded onto freshly glow discharged R 2/2 UltrAuFoil grids, prior to plunge freezing using a vitrobot MarkIV (ThermoFisher Scientific) with a blot force of 0 and 5 sec blot time at 100 % humidity and 22°C. Data collection used an FEI Titan Krios transmission electron microscope operated at 300 kV and equipped with a Gatan K3 direct detector and Gatan Quantum GIF energy filter, operated in zero-loss mode with a slit width of 20 eV. The dose rate was adjusted to 7 counts/pixel/s, and each movie was acquired in 80 frames of 50 ms. Automated data collection was carried out using Leginon^52^ at a nominal magnification of 105,000x with a pixel size of 0.835 Å. The nominal defocus values were set from −0.2 and −1.6 μm and a total of 9,729 movies were collected. Movie frame alignment, estimation of the microscope contrast-transfer function parameters, initial particle picking and extraction were carried out using Warp^55^.

Initially, we employed traditional single-particle image analysis to obtain cryo-EM structures of the UmbA4 filament observed in the presence of TUA polymers. First, the particles were extracted in a box covering sixteen UmbA4–TUA subunits and subjected to 2D classification prior to generating an *ab initio* reconstruction in cryoSPARC^53^. Then one round of 3D refinement was carried out with no imposed symmetry and the reconstructed map was used to estimate the initial helical symmetry parameters of the complex using Chimera^56^. This yielded initial estimates of 33.6 Å (rise) and 208.5° (twist ) which were used as a starting point for three-dimensional helical refinement with symmetry search in cryoSPARC without initial model. For subsequent picking of filament segments, micrographs were imported into cryoSPARC and the filament tracer module was used to pick filament segments from micrographs with a filament diameter of 80 Å and separation distance between segments of 0.4 x diameter with minimum and maximum filament diameter values for template-free tracing of 60 and 160 Å , respectively. 5,747,176 filament segments were extracted in 168 pixel (280.6 Å) boxes and subjected to reference-free 2D classification in cryoSPARC. Six well-defined class averages were selected for two rounds of reference-free *ab initio* reconstruction and heterogenous 3D refinements without imposing symmetry. 1,492,839 filament segments were selected and used for helical 3D refinement using cryoSPARC enabling non-uniform refinement, per-particle defocus refinement and refinement of helical parameters, yielding a reconstructions at 3.4 Å resolution. The selected dataset was transferred from cryoSPARC to Relion format using the pyem program package and particle images were subjected to the Bayesian polishing procedure implemented in Relion^57^ during which particles were re-extracted with a box size of 280 pixels and a pixel size of 1.0 Å. To eliminate poor quality particles, another round of reference-free 2D classification and *ab initio* reconstruction in cryoSPARC was used to re-classify the data and the generated models were used as references for heterogeneous 3D refinement. Helical symmetry search was then carried out again in cryoSPARC. The final helical 3D refinements of the TUA-bound UmbA4 complex structure was carried out using cryoSPARC enabling non-uniform refinement, per-particle defocus refinement and refinement of helical parameters, yielding a reconstruction at 3.4 Å resolution comprising 1,182,274 particles with a rise of 33.13 Å and a helical twist of 208.86°.

Local resolution estimation, filtering, and sharpening were carried out using cryoSPARC. Reported resolutions are based on the gold-standard Fourier shell correlation (FSC) of 0.143 criterion and Fourier shell correlation curves were corrected for the effects of soft masking by high-resolution noise substitution ^58,59^. The details of the image processing procedure are summarized in Figure S5E,K.

##### Model building and refinement

Initial models of UmbA4 and UmbB were obtained using AlphaFold3^60^. UCSF Chimera^56^ and Coot^61^ were used to fit atomic models into the cryo-EM maps. Carbohydrate models were built and refined into density using Rosetta ^62^. Building off previous carbohydrate modelling tools^62^, Rosetta parameter files for each of the five unique monosaccharides were manually built in Rosetta. An initial model was constructed using knowledge of the placement of the reducing end: the carbohydrate chain was grown by adding two sugars at a time and refining the growing model into density. With the initial carbohydrate model built, we then further refined the model of the complete assembly. Using the helical symmetry present in the cryo-EM map, we symmetrically refined both protein and carbohydrate together in the model using Rosetta^63^. The final model contains UmbA4 residues 33–705 and TUA glycans 1–12. The refinement statistics are summarized in Table S5.

#### Mutant UmbA4-containing Umb particle testing

Sup^Sc^ was prepared from *S. coelicolor ∆umbA4* strains expressing wild-type UmbA4, UmbA4(Y551A), UmbA4(N633A), or UmbA4(W668A) from pSET152 as described above. Sup^Sc^ was also prepared from *S. coelicolor ∆umbA4* with empty pSET152. All the supernatant samples were subjected to western blot analysis (β-His Antibody, QIAGEN), and UmbA4 band intensities were quantified using ImageJ^64^. Based on these results, all samples were diluted to contain equivalent concentrations of UmbA4 using supernatant generated from the empty plasmid-containing strain. Another western blot analysis was performed to confirm that UmbA4 concentrations were equal. All sup^Sc^ with normalized UmbA4 content and sup^Sc^ from the empty plasmid-containing strain were serially diluted 2-fold in PBS and assessed for toxicity against *S. griseus*.

*S. griseus* precultures were grown in 30 mL TSBY medium for 20 h, then back diluted to an OD_600_ of 0.01. 90-μL aliquots of the diluted culture were mixed with 10 μL of each sup^Sc^ dilution in a 96-well plate then incubated in a LogPhase for 10 h. Cell viability was assessed using BacTiter-Glo reagent.

#### Bioinformatics analysis

##### WTA and TUA biosynthesis loci identification

We compiled a protein database comprising 20 selected *Streptomyces* genomes, along with several non-*Streptomyces* genomes from other Actinobacteria as outgroups. We initiated multiple PSI-BLAST searches^65^ using several proteins in the Utr locus, and nearby WTA biosynthetic proteins including SGR_4957 (TagF), SGR_4943 (UtrC), SGR_4956 (TarIJ), and two adjacently encoded ribosomal proteins to use as locus placement anchors, SGR_4953 (Ribosomal protein L21p), and SGR_4954 (Ribosomal protein L27). These searches employed a stringent cutoff e-value of 0.005. For each identified homolog, we extracted and analyzed the surrounding genomic context, encompassing 15 upstream and 15 downstream neighboring genes. We found that they typically occupied the same genomic locus, and these were unique to each genome. Proteins recovered from these loci were systematically grouped into major clusters based on sequence similarity using the BLASTCLUST program (https://ftp.ncbi.nih.gov/blast/documents/blastclust.html). Subsequently, we annotated these protein clusters by identifying their constituent domains through hmmscan searches^66^ against Pfam^67^ and our curated domain profiles. Additionally, to predict the function of domain families that are not annotated, we generated the structural models using AlphaFold^60^ and identified the distant homologs using the DALI program^68^.

##### Streptomyces UmbA protein analysis

We initially compiled a comprehensive dataset consisting of 11,223 annotated *Streptomycetaceae* genomes retrieved from the NCBI GenBank database^69^, of which 10,618 belonged specifically to the genus *Streptomyces*. Using the inactive trypsin domain of UmbA (residues 21–230 of CAC36720.1) as a query, PSI-BLAST searches^65^ were conducted against this custom genome database with a stringent E-value cutoff of 0.005. This search yielded 9,455 sequences harboring the inactive trypsin domain (UmbA homologs).

Domain architecture annotations were subsequently performed using the hmmscan program^66^, querying both the Pfam profile database^67^ and a custom-curated domain profile database. This analysis highlighted a significant association between the inactive trypsin domain and various membrane-localization domains positioned in the C-terminal regions, including diverse β-propeller domains, ricinB-lectin, and bulb-lectin domains. To further analyze these membrane-localization regions, we extracted the corresponding C-terminal sequences larger than 20 amino acids, resulting in a dataset of 9192 sequences (Dataset 1). Additionally, we observed instances in which the inactive trypsin domain (UmbA) and the associated lectin domains reside in separate but neighboring genes. To systematically retrieve these occurrences, neighboring genes (five upstream and five downstream) relative to each UmbA-containing gene were extracted. These neighboring sequences were subjected to similarity searches against the previously extracted C-terminal UmbA regions using BLASTP. Sequences displaying significant similarity to any of these regions were retained, resulting in an additional set of 667 sequences (Dataset 2). Combining these two datasets resulted in a consolidated dataset comprising 9,859 unique sequences.

To achieve a comprehensive identification and classification of domain families within these 9,859 sequences, we employed the following strategy:

1. Network clustering analysis was conducted using the CLANS program^70^, which employs the Fruchterman and Reingold force-directed layout algorithm based on significant high-scoring segment pairs (HSPs) identified through all-against-all BLASTP searches (P-value: 0.0001). The sequences, represented as nodes, and the edges between are attractive forces which are proportional to the negative logarithm of the alignment significance values. This approach facilitated the identification of major sequence clusters among these 9,859 sequences.
2. For each densely connected subgraph (cluster), multiple sequence alignment was generated using either KALIGN^71^ or MUSCLE^72^. Representative sequences from each alignment were selected for structural modeling using AlphaFold^60,73^. Domain boundaries were delineated through analyses of inter-residue distance matrices generated from predicted structural models.
3. The identified domain sequences were further clustered using the CLANS program to precisely define distinct domain families, yielding a total of 41 major families.
4. These domain families were subsequently grouped into major fold superfamilies through sequence profile comparisons using HHsearch^74^ and structural similarity comparisons using the DALI server^68^.

Through this comprehensive analysis, we identified 41 distinct domain families that belong to 12 overarching fold superfamilies. These include 12 families of five-bladed β-propellers (5BP1 to 5BP12), four families of six-bladed β-propellers (6BP1 to 6BP4), seven families of seven-bladed β-propellers (7BP1 to 7BP7), nine ricinB-lectin families (RB1 to RB9), one bulb-lectin family, one jellyroll-like fold family, two GDSL hydrolase domain families, one laminin_G_3 family, one GDPD domain family, one CBM_4_9 family, one newly identified immunoglobulin-like domain family (BPA-Ig), and a novel β-sandwich fold family designated TAC4.

##### UmbC protein distribution analysis

To assess the complement of UmbC proteins encoded within the genomes of UmbA-containing *Streptomycetae* genomes, we searched the same set of genomes by scanning for genes encoding ALF repeats and toxin domain profiles. This led to the identification of 8131 UmbC, among which 7099 (87.3%) are annotated with toxin domains (Table S6). UmbC proteins lacking the full complement of eight ALF repeats or lacking a recognizable C-terminal toxin domain were filtered from the dataset. The number of UmbC and UmbA proteins encoded by individual *Streptomyces* strains were grouped and counted. The strains were then classified by species, and within each species, the strains encoding the highest number of UmbA proteins were selected as the representative. The UmbA and UmbC protein counts for these representative strains were further analyzed and are shown in Figure 6A,B.

### QUANTIFICATION AND STATISTICAL ANALYSIS

Significance of differences in growth yields from supernatant toxicity assays and in protein abundances from whole cell proteome analyses were determined using one-way ANOVA with Dunnett’s multiple comparisons test. Tests were performed using Graphpad Prism. For bacterial growth assays, bacterial pull-down assays, and cell binding assays, the number of replicates collected from independent cultures grown in parallel on a single day are indicated in corresponding methods or figure legends. Each experiment was also replicated at least once on separate days with at least two additional cultures. Statistical methods were not used to predetermine sample size, and randomization and blinding were not employed.

## Supplementary Material

1 **Figure S3. Identification and ALE of a target of *S. griseus* umbrella toxin particle, related to** Figure 3. A) Loci encoding Umb protein complex components in *S. griseus*. UmbA is encoded distantly from other complex components. B) Schematic illustrating the composition of the single umbrella toxin particle produced by *S. griseus.* Toxin domain labeled according to predicted RNase activity. C) *S. griseus* Umb toxin susceptibility screening results. Z-scores calculated from ratio of growth in control supernatant to growth in sup^Sg^ from two biological replicates of the screen; scores >2 indicate significant Umb-dependent inhibition. Raw data provided in Table S1. D) Results of screen for sup^Sg^ resistance among evolved isolates from passages 3–5 of *S. antibioticus* ALE. Grids represent 96-well plates used to grow isolates for 16 h with sup^Sg^ treatment. Numbers indicate the ratio of evolved isolate growth to that of the wild-type ancestral strain. Isolates with a growth ratio exceeding 3 were considered resistant to sup^Sg^ and are illustrated by shaded wells. E) Schematic indicating the location of mutations within the *utr* carbohydrate biosynthesis gene cluster of *S. antibioticus* selected during ALE with sup^Sg^ treatment. Numbers represent the number of times the indicated mutation was observed across six isolates sequenced. Genes are colored according to orthology with Figure 3C.

2 Document S1. Table S1 and S5.

3 Table S2. Mutations detected in *S. griseus* and *S. antibioticus* clones obtained following ALE in the presence of *S. coelicolor and S. griseus* umbrella toxins, respectively, related to Figure 2 and Figure 3.

4 Table S3. Bioinformatic analysis of proteins encoded by the *S. griseus utr* operon, related to Figure 2.

5 Table S4. Chemical shift assignments of carbohydrate and polyol*P* residues in wild-type and ∆*utrC* preparations of TUA–WTA, TUA and WTA from S. *griseus* and *S. antibioticus*, respectively, related to Figure 2 and Figure 3.

6

7 Table S6. Diversity of UmbA lectin domains and UmbC toxin domains within Streptomycetaceae family, related to Figure 6.

8 Table S7. Primers used in this study, related to Key Resources Table.

9 **Figure S1. Isolation of *S. griseus* mutants resistant to sup^Sc^ via adaptive laboratory evolution, related to** Figure 2. A) Growth of *S. griseus* after 16 h of treatment with wild-type sup^Sc^ serially diluted in *∆umbC2* sup^Sc^. The concentration of wild-type sup^Sc^ in the treatment mixture is indicated. Data are normalized by the maximum and minimum levels of growth (measured as relative luminescence units (r.l.u.)), corresponding to treatment with only *∆umbC2* or wild-type sup^Sc^, respectively. Data represent mean ± s.d. (*n* = 3). B) Results of screen for sup^Sc^ resistance among evolved isolates from passages 1–3 of *S. griseus* ALE. Grids represent 96-well plates used to grow isolates for 16 h with sup^Sc^ treatment. Numbers indicate the ratio of evolved isolate growth to that of the wild-type ancestral strain. Isolates with a growth ratio exceeding 3 were considered resistant to sup^Sc^ and are illustrated by shaded wells. C) Predicted structure of UtrC (SGR_4943) compared to the crystal structure of TarS^17^. The magnified inset panel shows an overlay of the predicted catalytic domain of UtrC with the experimentally characterized catalytic domain of TarS, including the catalytic residue D181.

10 **Figure S2. NMR-based structural analysis of wild-type *S. griseus* TUA–WTA, related to** Figure 2. A) NMR structural analysis of TUA–WTA total TCA hydrolysate from *S. griseus*. Top right, 2D multiplicity-edited ^1^H,^13^C-HSQC NMR spectrum with carbohydrate signals labeled using residue codes from panel (D). Signals of glycosidically linked positions are labeled in bold. For clarity, polyol*P* signals of the WTA were not labeled. Positive signals (CH groups) are drawn in black and negative ones (CH_2_) in grey. Bottom right, a region of ^1^H,^1^H-ROESY spectrum that contains through-space correlation signals originating from the anomeric hydrogens, as well as H-4 of the acetylated residue D. Signal labels consist of the residue code and the ring position number of each of the two interacting nuclei. E.g., A1–2 marks signal due to correlation between H-1 and H-2 in residue A. Correlations between two residues across the glycosidic bond are labeled in bold (e.g., G1-Rg4). Bottom left, anomeric region of the ^1^H,^13^C-HSQC spectrum. Top left, an overlay of ^1^H,^13^C-HMBC (black signals) and ^1^H,^13^C-HSQC (olive signal) spectra in the ^13^C region up-field from the anomeric signals shown in the panel below. The HMBC signals show correlations between the anomeric hydrogen and carbon-2, −3 and/or −5 within a residue (e.g., A3–1). Further, the HMBC signals include inter-residue correlations through glycosidic bonds between anomeric hydrogen and the closest carbon in the aglycon (labeled in bold, e.g., G4-A1). B) 2D multiplicity-edited ^1^H,^13^C-HSQC NMR spectra of the larger TUA–WTA and the smaller WTA obtained from SEC of the total wild-type *S. griseus* cell wall TCA hydrolysate (Figure 2F). Signal assignments are based on the analysis of a full set of 2D NMR experiments acquired for each sample. Signals of TUA carbohydrate and the linking Rbo-5*P* are labeled in brown, while the signals of WTA polyol*P* residues in blue. Positive signals (CH and CH_3_) are shown in red and negative signals (CH_2_) in blue. C) 2D multiplicity-edited ^1^H,^13^C-HSQC NMR spectrum of WT *S. griseus* dephosphorylated TUA obtained by SEC (Figure 2F). Signal assignments based on the analysis of a full set of 2D NMR experiments are indicated. D) Schematic structure of the TUA with NMR assignment code indicated in brown for each residue. The codes are as in panels A-C, and in Table S4. E) Major polyol-*P* units identified in the WTA, with NMR assignment codes indicated in blue.

11 **Figure S4. Structural analysis of *S. antibioticus* cell wall polymers, related to** Figure 3. A) 2D multiplicity-edited ^1^H,^13^C-HSQC NMR spectra of *S. antibioticus* wild-type (left) and Δ*utrC* (right) intact polymers isolated from cell walls by acid hydrolysis. The corresponding 1D ^1^H spectra are shown above each HSQC panel. Signals of the TUA are labeled in brown and signals of WTA in blue. The labels show the residue code pertinent to the TUA and WTA structures in panels (C) and (D), respectively, and C–H group number in that residue. Positive signals (CH and CH_3_) are shown in red and negative signals (CH_2_) in blue. Asterisks mark weak COSY-type peaks that formed between the strong signals of residue A. B) Expanded anomeric region of the same HSQC spectra shown in panel (A). Signal and spectra colors have the same meaning as in panel (A). C) Structure of the TUA oligomer with a repeating unit consisting of a single →4)-GlcNAc3NAcA-β(1→ residue. The TUA is terminated with a 4O-acetylated GlcNAc3NAcA residue on the non-reducing end, while the reducing-end residue of GlcNAc3NAcA forms a β(1→4) glycosidic bond with a ribitol-5-phosphate residue. D) Ribitol phosphate units identified in the wild-type and mutant WTA. The WTA contains 1,5-linked ribitol-*P* residues, a large portion of which is substituted by β-Glc residue in the 2- or 4-position. The mutant WTA has much higher content of a terminal ribitol-5*P* residue (residue Rc, in bold), presumably equivalent to the terminal Rbo-5P that is substituted by TUA in the wild-type material. E) ^1^H NMR (top) and DOSY (bottom) spectra of the *S. antibioticus* wild-type (left) and Δ*utrC* (right) TUA/WTA preparations. The wild-type DOSY spectrum shows that the TUA and WTA have the same diffusion coefficient, indicating the same size. All WTA signals in the Δ*utrC* DOSY are also aligned at the same *D* level, consistent with a size-uniform preparation. Given its lower *D*, the size of Δ*utrC* WTA is likely somewhat larger compared to the wild-type TUA-WTA that exhibits a larger *D*. Gray, green and blue backgrounds mark regions with TUA only, WTA only and overlapping TUA+WTA signals, respectively.

12 **Figure S5. Cryo-EM data processing of the UmbA4 and TUA molecules bound UmbA4 complex datasets, related to** Figure 5. A, B, F and G) Representative electron micrographs (A, F) and 2D class averages (B, G) of the UmbA4 complex (A, B) or TUA molecules bound UmbA4 complex (F, G) embedded in vitreous ice. Scale bars: 100 nm (A,F), 20 nm (B,G). C,H) Gold-standard Fourier shell correlation curve of the UmbA4 complex (C) or TUA molecules bound UmbA4 complex (H). The 0.143 cutoff is indicated by a horizontal dashed line. The angular distribution of particle images calculated using cryoSPARC is shown as a heat map below. D) 3D reconstruction of UmbA4 complex that encompassed two proteins (UmbA4 in blue and UmbB in magenta). E,K) Data processing flowchart. CTF: contrast transfer function; NUR: non-uniform refinement. J) Local resolution estimation of TUA molecules bound UmbA4 complex reconstruction calculated using cryoSPARC and plotted on the sharpened maps. I) Unsharpened (transparent) and sharpened (opaque) cryo-EM maps of a helical segment of the TUA–UmbA4–UmbB1–3 complex. Regions encompassing TUA and UmbA4 are colored based on model proximity.

13 **Figure S6. TUA binds at conserved sites on the β-propeller lectin, related to** Figure 5. A) Overview of sites I, II, and III on the asymmetric unit containing three UmbA4 protomers. B) Close-up of site III. TUA packs between UmbA4(2) and UmbA4(3) making weak contacts with both protomers. C) Sequence alignment of the β-propeller lectin blades. Secondary structure is indicated above the alignment. Residues that form direct interactions with TUA are indicated (red). Residue positions that interact with TUA in both site I and II are indicated (circle). D) Top-down view of a single UmbA4 molecule indicating sites that make contact with TUA in different protomers in the cryo-EM structure. Individual blades of the β-propeller are labeled (b1–6). E, F) Zoom-in view of UmbA4–TUA interaction sites I (E) and II (F), indicating the contacting amino acids.

## Figures and Tables

**Table:** Key resources table

REAGENT or RESOURCE	SOURCE	IDENTIFIER
Antibodies		
Mouse monoclonal anti-His antibodies conjugated to horseradish peroxidase	QIAGEN	Cat#34460
Bacterial and virus strains		
*Escherichia coli* DH5α	Thermo Fisher Scientific	Cat#18258012
*E. coli* ET12567(pUZ8002)	Sangon Biotech	Cat#S0052
*Streptomyces coelicolor* A3(2)	ARS Culture Collection	NRRL No. B-3062
*S. coelicolor ∆umbC1–3*	This study	N/A
*S. coelicolor ∆umbC1*	Zhao et al.^16^	N/A
*S. coelicolor ∆umbC2*	Zhao et al.^16^	N/A
*S. coelicolor ∆umbC3*	Zhao et al.^16^	N/A
*S. coelicolor ∆umbC1 ∆umbC2*	This study	N/A
*S. coelicolor ∆umbC1 ∆umbC3*	This study	N/A
*S. coelicolor ∆umbC2 ∆umbC3*	This study	N/A
*S. coelicolor ∆umbA1*	This study	N/A
*S. coelicolor ∆umbA2*	This study	N/A
*S. coelicolor ∆umbA3*	This study	N/A
*S. coelicolor ∆umbA4*	This study	N/A
*S. coelicolor ∆umbA5*	This study	N/A
*S. coelicolor ∆umbA6*	This study	N/A
*S. coelicolor ∆umbA1 ∆umbA6*	This study	N/A
*S. coelicolor ∆umbC1–3 attB*::pSET152–*umbA4*–2xGGGGS–3xK–6xH	This study	N/A
*S. coelicolor ∆umbA4 attB*::pSET152	This study	N/A
*S. coelicolor ∆umbA4 attB*::pSET152–*umbA4*–2xGGGGS–3xK–6xH	This study	N/A
*S. coelicolor ∆umbA4 attB*::pSET152–*umbA4*(Y551A)–2xGGGGS–3xK–6xH	This study	N/A
*S. coelicolor ∆umbA4 attB*::pSET152–*umbA4*(N633A)–2xGGGGS–3xK–6xH	This study	N/A
*S. coelicolor ∆umbA4 attB*::pSET152–*umbA4*(W668A)–2xGGGGS–3xK–6xH	This study	N/A
*S. coelicolor attB*::pSET152	This study	N/A
*S. coelicolor attB*::pSET152–*umbA1*^*Sc*^	This study	N/A
*S. coelicolor attB*::pSET152–*umbA1*(T)^*Sc*^*–umbA*(L)^*Sg*^	This study	N/A
*S. coelicolor attB*::pSET152–*umbA4*^*Sc*^	This study	N/A
*S. coelicolor attB*::pSET152–*umbA4*(T)^*Sc*^*–umbA*(L)^*Sg*^	This study	N/A
*S. coelicolor ∆umbC1–3 attB*::pSET152–*umbA1*(T)^*Sc*^*–umbA*(L)^*Sg*^	This study	N/A
*S. coelicolor ∆umbC1 attB*::pSET152–*umbA1*(T)^*Sc*^*–umbA*(L)^*Sg*^	This study	N/A
*S. coelicolor ∆umbC2 attB*::pSET152–*umbA1*(T)^*Sc*^*–umbA*(L)^*Sg*^	This study	N/A
*S. coelicolor ∆umbC3 attB*::pSET152–*umbA1*(T)^*Sc*^*–umbA*(L)^*Sg*^	This study	N/A
*S. coelicolor ∆umbC1–3 attB*::pSET152–*umbA4*(T)^*Sc*^*–umbA*(L)^*Sg*^	This study	N/A
*S. coelicolor ∆umbC1 attB*::pSET152–*umbA4*(T)^*Sc*^*–umbA*(L)^*Sg*^	This study	N/A
*S. coelicolor ∆umbC2 attB*::pSET152–*umbA4*(T)^*Sc*^*–umbA*(L)^*Sg*^	This study	N/A
*S. coelicolor ∆umbC3 attB*::pSET152–*umbA4*(T)^*Sc*^*–umbA*(L)^*Sg*^	This study	N/A
*S. coelicolor ∆umbC1 ∆umbC2 attB*::pSET152–*umbA4*(T)^*Sc*^*–umbA*(L)^*Sg*^	This study	N/A
*S. coelicolor ∆umbC1 ∆umbC3 attB*::pSET152–*umbA4*(T)^*Sc*^*–umbA*(L)^*Sg*^	This study	N/A
*S. coelicolor ∆umbC2 ∆umbC3 attB*::pSET152–*umbA4*(T)^*Sc*^*–umbA*(L)^*Sg*^	This study	N/A
*Streptomyces griseus*	ARS Culture Collection	NRRL B-2682
*S. griseus ∆utrC* ^*Sg*^	This study	N/A
*S. griseus ∆utrC attB::*pSET152*–utrC*^*Sg*^	This study	N/A
*S. griseus attB::*pSET152–*utrC*^*Sg*^(D181N)	This study	N/A
*S. griseus ∆umbC* ^*Sg*^	This study	N/A
*Streptomyces achromogenes* SANT-13	This study	N/A
*Streptomyces ambofaciens* SAI_195	Zhao et al.^16^	N/A
*Streptomyces antibioticus*	ARS Culture Collection	NRRL B-1701
*S. antibioticus ∆utrC* ^*Sa*^	This study	N/A
*S. antibioticus ∆utrG* ^*Sa*^	This study	N/A
Biological samples		
Chemicals, peptides, and recombinant proteins		
5-Fluorocytosine	TCI Chemicals	Cat#F0321
5-Bromo-4-chloro-3-indolyl-β-d-glucuronide	Sigma-Aldrich	Cat#B5285
Apramycin	Sigma-Aldrich	Cat#A2024
Kanamycin	Goldbio	Cat#K-120–50
Chloramphenicol	Fisher Bioreagents	Cat#BP904–100
Trimethoprim	Sigma-Aldrich	Cat#T7883–5G
Dithiothreitol	Research Products International	Cat#D11000–25.0
Sequencing grade modified trypsin	Promega	Cat#V5111
Trifluoroacetic acid	Sigma-Aldrich	Cat#80457–10ML
Iodoacetamide	Thermo Scientific	Cat#A39271
Trichloroacetic acid	Sigma-Aldrich	Cat#T6399–500G
Hydrofluoric acid	Fisher Chemical	Cat#A513500
Super-DHB	Sigma-Aldrich	Cat#50862–1G-F
D_2_O	Sigma-Aldrich	Cat#151882
DSS-*d*_6_	Cambridge Isotope Laboratories	Cat#DLM-8206-PK
D_2_O	Thermo Scientific	Cat#320700075
Wall teichoic acid—teichuronic acid	This study	N/A
Teichuronic acid	This study	N/A
Tetramethylrhodamine	Cayman Chemical Company	Cat#19593
Critical commercial assays		
Gibson assembly kit	New England Biolabs	Cat#E2611L
InstaGene Matrix	Bio-Rad	Cat#732–6030
BacTiter-Glo Microbial Cell Viability Assay	Promega	Cat#G8231
Pierce™ BCA Protein Assay Kit	Thermo Scientific	Cat#23225
Zymo 1X DNA/RNA Shield	Zymo Research	Cat#R1100–50
DNeasy Blood & Tissue kit	Qiagen	Cat#69506
Nextera DNA Flex Library Prep Kit	Illumina	Cat#20018705
Deposited data		
The complete genome sequences of *S. antibioticus* NRRL B-1701	This study	BioSample: SAMN48121715
Whole genome sequence of *S. griseus* and *S.* *antibioticus* isolates evolved for resistance to umbrella particles	This study	BioProject: PRJNA1254743
Experimental models: Cell lines		
Experimental models: Organisms/strains		
Oligonucleotides		
See Table S7		
Recombinant DNA		
pKGLP2a	Zhao et al.^16^	N/A
pKGLP2a–*codA(sm)*	This study	N/A
pKGLP2a–*codA(sm)*::*∆utrC*^*Sg*^	This study	N/A
pKGLP2a–∆*umbA1*	This study	N/A
pKGLP2a–∆*umbA2*	This study	N/A
pKGLP2a–∆*umbA3*	This study	N/A
pKGLP2a–∆*umbA4*	This study	N/A
pKGLP2a–∆*umbA5*	This study	N/A
pKGLP2a–∆*umbA6*	This study	N/A
pKGLP2a–∆*umbC1*	Zhao et al.^16^	N/A
pKGLP2a–∆*umbC2*	Zhao et al.^16^	N/A
pKGLP2a–∆*umbC3*	Zhao et al.^16^	N/A
pKGLP2a–*codA(sm)*–*∆umbC*^*Sg*^	This study	N/A
pKGLP2a–*codA(sm)*–*∆utrC*^*Sa*^	This study	N/A
pKGLP2a–*codA(sm)*–*∆utrG*^*Sa*^	This study	N/A
pSET152	Bierman et al.^45^	N/A
pSET152–*utrC*^*Sg*^	This study	N/A
pSET152–*utrC*^*Sg*^(D181N)	This study	N/A
pSET152–*umbA4*–2×GGGGS–3×K–6×H	This study	N/A
pSET152–*umbA4*(Y551A)–2×GGGGS–3×K–6×H	This study	N/A
pSET152–*umbA4*(N633A)–2×GGGGS–3×K–6×H	This study	N/A
pSET152–*umbA4*(W668A)–2×GGGGS–3×K–6×H	This study	N/A
pSET152–*umbA1*^*Sc*^	This study	N/A
pSET152–*umbA1*(T)^*Sc*^–*umbA*(L)^Sg^	This study	N/A
pSET152–*umbA4*^*Sc*^	This study	N/A
pSET152–*umbA4*(T)^*Sc*^–*umbA*(L)^Sg^	This study	N/A
Software and algorithms		
MaxQuant	Tyanova et al.^48^	https://www.biochem.mpg.de/6304115/maxquant RRID:SCR_014485
Geneious Prime 2023.1.2	Geneious, software	https://www.geneious.com; RRID:SCR_010519
Graphpad Prism 9	GraphPad, software	https://www.graphpad.com; RRID:SCR_022798
Adobe Illustrator 27.3.1	Adobe Systems Incorporated, software	https://www.adobe.com/products/illustrator; RRID:SCR_010279
Topspin 4.0.5	BRUKER, software	https://www.bruker.com/en/products-and-solutions/magnetic-resonance/nmr-software/topspin.html RRID:SCR_014227
AnalysisAssign 3.2.0	Skinner et al.^49^	https://ccpn.ac.uk/software/downloads/
MestReNova (14.2.3)	Mestrelab, software	https://mestrelab.com/download
Warp v1.1.0 beta	Tegunov et al.^55^	https://github.com/cramerlab/warp
UCSF ChimeraX v1.8	Pettersen et al.^56^	https://www.cgl.ucsf.edu/chimerax/;
Relion v5.0	Zivanov et al.^57^	http://www2.mrc-lmb.cam.ac.uk/relion
AlphaFold3	Abramson et al.^60^	https://alphafoldserver.com/
Coot v0.9.8.95EL	Emsley et al.^61^	http://www2.mrc-lmb.cam.ac.uk/personal/pemsley/coot/
Rosetta	Frenz et al.^62^	https://rosettacommons.org/software/
cryoSPARC v4.4.0	Punjani et al.^53^	https://cryosparc.com/
BLASTCLUST program		https://ftp.ncbi.nih.gov/blast/documents/blastclust.html
PSI-BLAST	Altschul et al.^65^	
hmmscan program	Eddy et al.^66^	http://hmmer.janelia.org/ SCR_005305
pfam	Mistry et al.^67^	http://pfam.xfam.org/ RRID:SCR_004726
DALI program	Holm et al.^68^	http://ekhidna.biocenter.helsinki.fi/dali_server RRID:SCR_013433
CLANS program	Frickey et al.^70^	https://toolkit.tuebingen.mpg.de/tools/clans
KALIGN	Lassmann et al.^71^	http://www.ebi.ac.uk/Tools/msa/kalign/ RRID:SCR_011810
MUSCLE	Edgar et al.^72^	http://www.ebi.ac.uk/Tools/msa/muscle/ RRID:SCR_011812
HHsearch	Soding et al.^74^	https://github.com/soedinglab/hh-suite RRID:SCR_016133
ImageJ	Schneider et al.^64^	https://imagej.net/ RRID:SCR_003070
Other		
Econo-Pac 10DG desalting column	Bio-Rad	Cat#7322010
Empore™ styrene divinyl benzene (SDB-RPS) extraction disks	Sigma-Aldrich	Cat#66886-U
TALON cobalt resin	Takara Bio	Cat#635503
PD SpinTrap G-25 column	Cytiva	Cat#28–9180-04
Nunc MicroWell 96-Well Optical-Bottom Plates	Fisher Scientific	Cat#1256635
UltrAuFoil grids	EMS	Cat#Q250AR2A
