# Supplementary figures and images for: The unique architecture of umbrella toxins permits a two-tiered molecular bet hedging strategy for interbacterial antagonism
Source: Cell. Author manuscript; Available in PMC 2026 Jun 17. (PMC13274773; doi:10.1016/j.cell.2025.10.044)

Figure S3

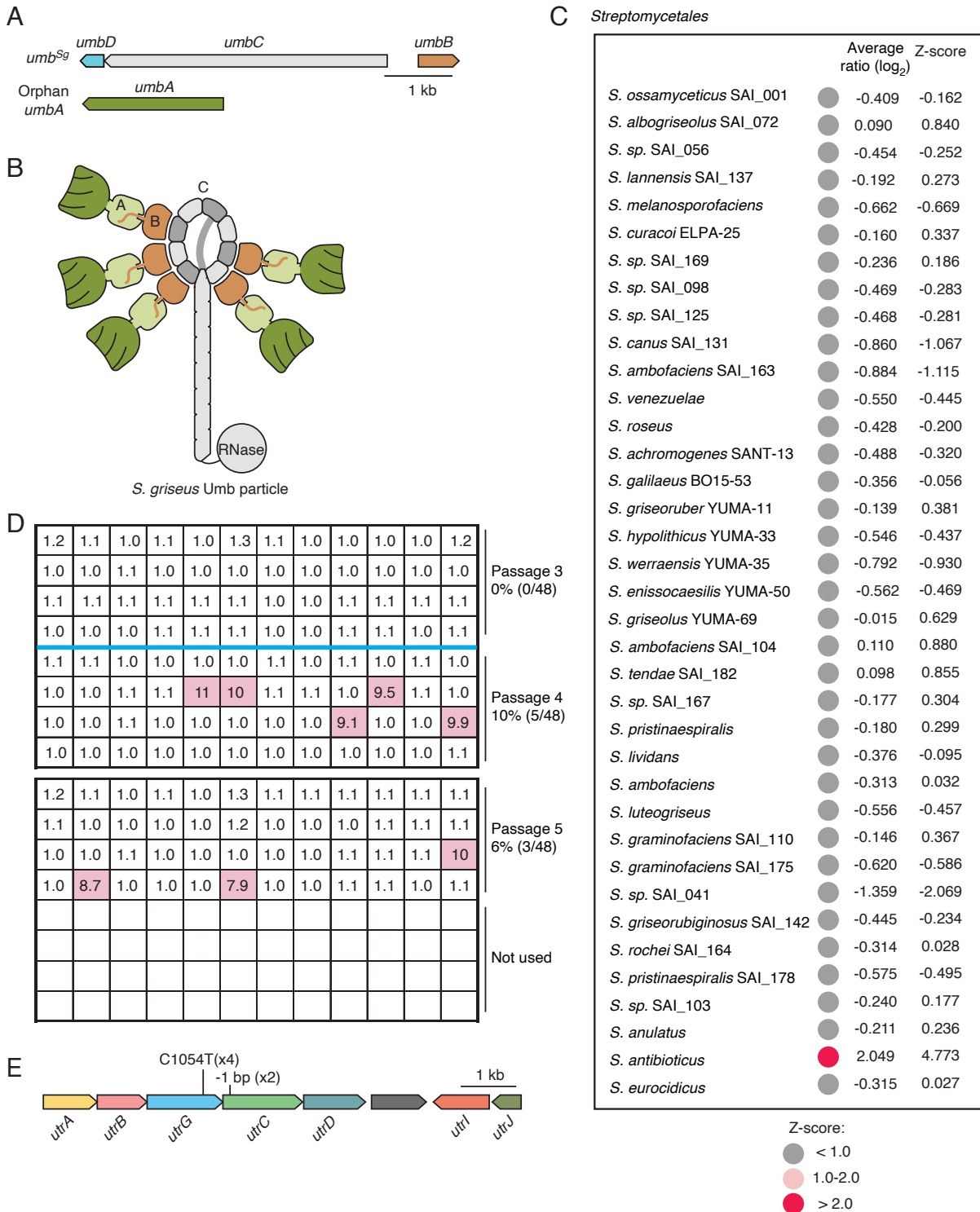

Supplement: 1 — Figure S3. Identification and ALE of a target of S. griseus umbrella toxin particle, related to Figure 3. A) Loci encoding Umb protein complex components in S. griseus. UmbA is encoded distantly from other complex components. B) Schematic illustrating the composition of the single umbrella toxin particle produced by S. griseus. Toxin domain labeled according to predicted RNase activity. C) S. griseus Umb toxin susceptibility screening results. Z-scores calculated from ratio of growth in control supernatant to growth in supSg from two biological replicates of the screen; scores >2 indicate significant Umb-dependent inhibition. Raw data provided in Table S1. D) Results of screen for supSg resistance among evolved isolates from passages 3–5 of S. antibioticus ALE. Grids represent 96-well plates used to grow isolates for 16 h with supSg treatment. Numbers indicate the ratio of evolved isolate growth to that of the wild-type ancestral strain. Isolates with a growth ratio exceeding 3 were considered resistant to supSg and are illustrated by shaded wells. E) Schematic indicating the location of mutations within the utr carbohydrate biosynthesis gene cluster of S. antibioticus selected during ALE with supSg treatment. Numbers represent the number of times the indicated mutation was observed across six isolates sequenced. Genes are colored according to orthology with Figure 3C. [file NIHMS2121434-supplement-1.pdf]

Figure S1

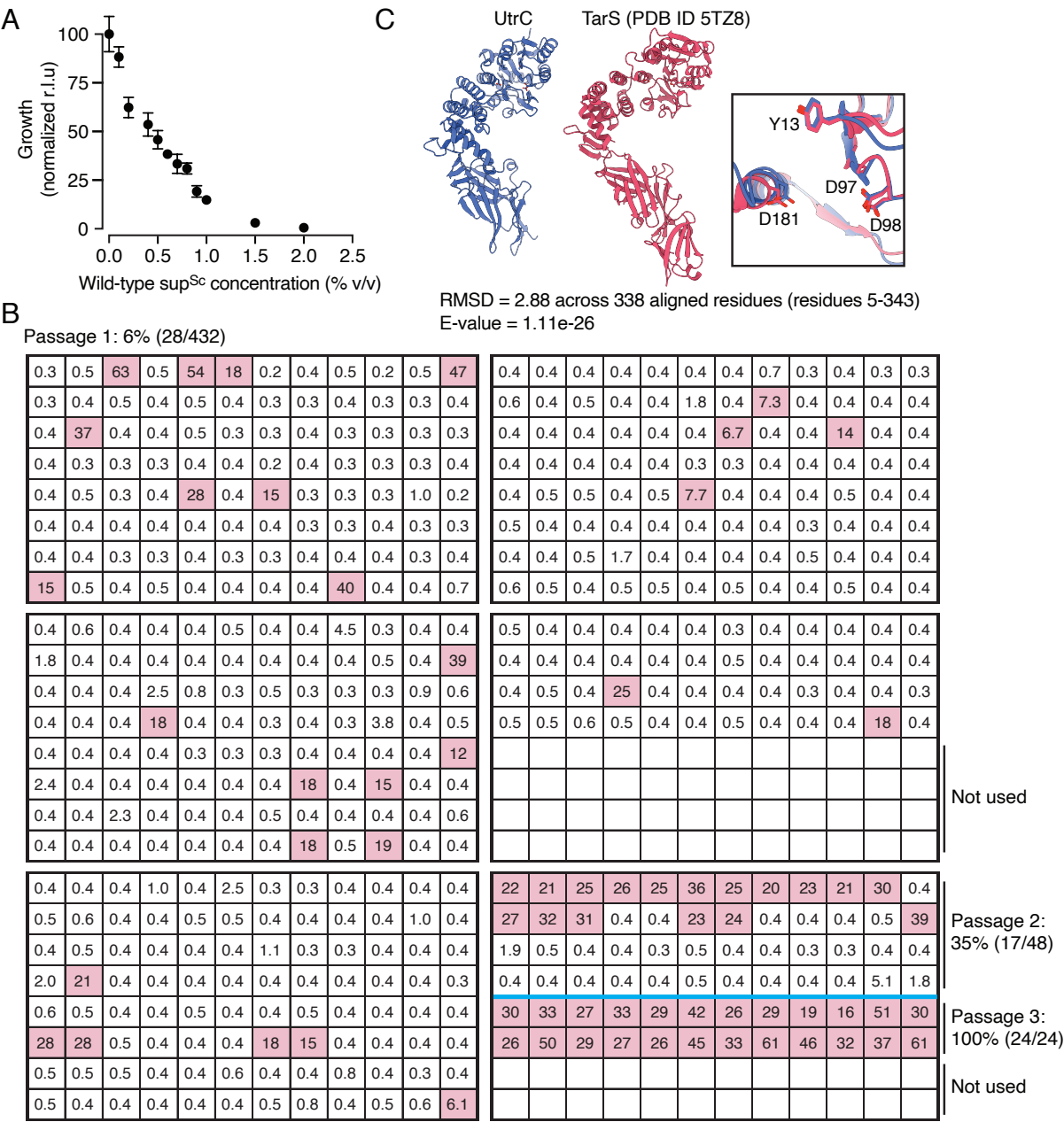

Supplement: 9 — Figure S1. Isolation of S. griseus mutants resistant to supSc via adaptive laboratory evolution, related to Figure 2. A) Growth of S. griseus after 16 h of treatment with wild-type supSc serially diluted in ∆umbC2 supSc. The concentration of wild-type supSc in the treatment mixture is indicated. Data are normalized by the maximum and minimum levels of growth (measured as relative luminescence units (r.l.u.)), corresponding to treatment with only ∆umbC2 or wild-type supSc, respectively. Data represent mean ± s.d. (n = 3). B) Results of screen for supSc resistance among evolved isolates from passages 1–3 of S. griseus ALE. Grids represent 96-well plates used to grow isolates for 16 h with supSc treatment. Numbers indicate the ratio of evolved isolate growth to that of the wild-type ancestral strain. Isolates with a growth ratio exceeding 3 were considered resistant to supSc and are illustrated by shaded wells. C) Predicted structure of UtrC (SGR_4943) compared to the crystal structure of TarS17. The magnified inset panel shows an overlay of the predicted catalytic domain of UtrC with the experimentally characterized catalytic domain of TarS, including the catalytic residue D181. [file NIHMS2121434-supplement-9.pdf]

Figure S2

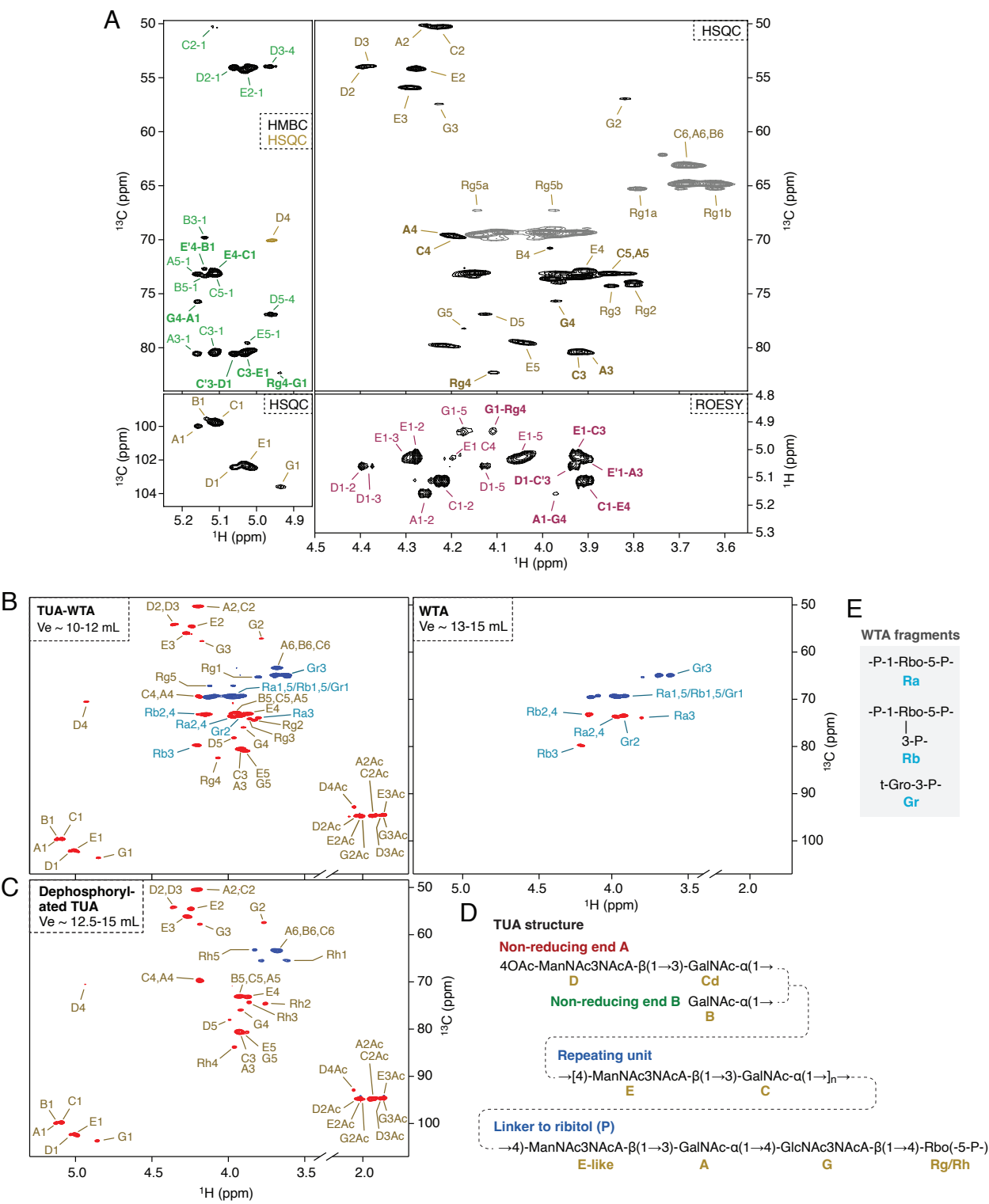

Supplement: 10 — Figure S2. NMR-based structural analysis of wild-type S. griseus TUA–WTA, related to Figure 2. A) NMR structural analysis of TUA–WTA total TCA hydrolysate from S. griseus. Top right, 2D multiplicity-edited 1H,13C-HSQC NMR spectrum with carbohydrate signals labeled using residue codes from panel (D). Signals of glycosidically linked positions are labeled in bold. For clarity, polyolP signals of the WTA were not labeled. Positive signals (CH groups) are drawn in black and negative ones (CH2) in grey. Bottom right, a region of 1H,1H-ROESY spectrum that contains through-space correlation signals originating from the anomeric hydrogens, as well as H-4 of the acetylated residue D. Signal labels consist of the residue code and the ring position number of each of the two interacting nuclei. E.g., A1–2 marks signal due to correlation between H-1 and H-2 in residue A. Correlations between two residues across the glycosidic bond are labeled in bold (e.g., G1-Rg4). Bottom left, anomeric region of the 1H,13C-HSQC spectrum. Top left, an overlay of 1H,13C-HMBC (black signals) and 1H,13C-HSQC (olive signal) spectra in the 13C region up-field from the anomeric signals shown in the panel below. The HMBC signals show correlations between the anomeric hydrogen and carbon-2, −3 and/or −5 within a residue (e.g., A3–1). Further, the HMBC signals include inter-residue correlations through glycosidic bonds between anomeric hydrogen and the closest carbon in the aglycon (labeled in bold, e.g., G4-A1). B) 2D multiplicity-edited 1H,13C-HSQC NMR spectra of the larger TUA–WTA and the smaller WTA obtained from SEC of the total wild-type S. griseus cell wall TCA hydrolysate (Figure 2F). Signal assignments are based on the analysis of a full set of 2D NMR experiments acquired for each sample. Signals of TUA carbohydrate and the linking Rbo-5P are labeled in brown, while the signals of WTA polyolP residues in blue. Positive signals (CH and CH3) are shown in red and negative signals (CH2) in blue. C) [file NIHMS2121434-supplement-10.pdf]

Figure S4

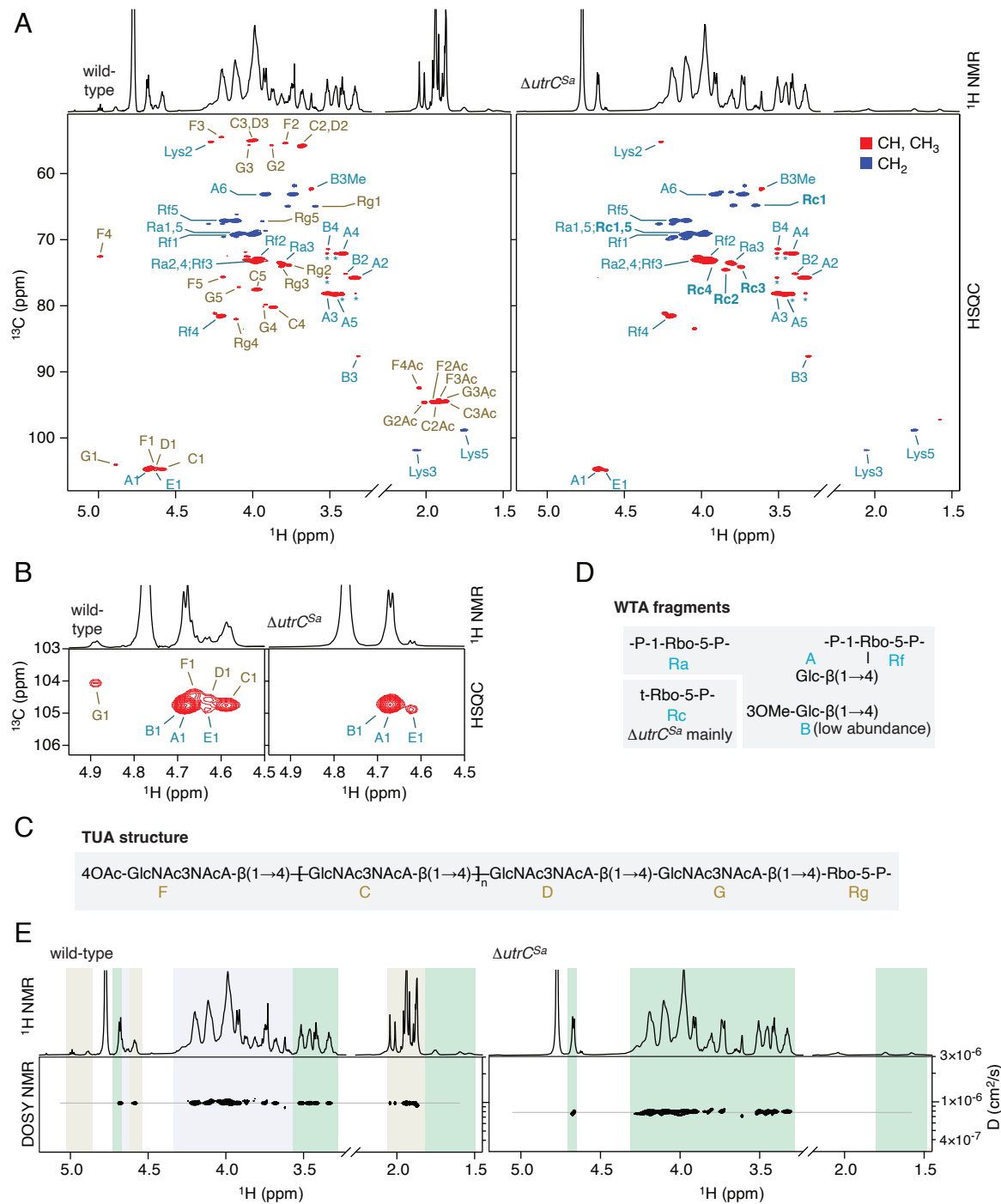

Supplement: 11 — Figure S4. Structural analysis of S. antibioticus cell wall polymers, related to Figure 3. A) 2D multiplicity-edited 1H,13C-HSQC NMR spectra of S. antibioticus wild-type (left) and ΔutrC (right) intact polymers isolated from cell walls by acid hydrolysis. The corresponding 1D 1H spectra are shown above each HSQC panel. Signals of the TUA are labeled in brown and signals of WTA in blue. The labels show the residue code pertinent to the TUA and WTA structures in panels (C) and (D), respectively, and C–H group number in that residue. Positive signals (CH and CH3) are shown in red and negative signals (CH2) in blue. Asterisks mark weak COSY-type peaks that formed between the strong signals of residue A. B) Expanded anomeric region of the same HSQC spectra shown in panel (A). Signal and spectra colors have the same meaning as in panel (A). C) Structure of the TUA oligomer with a repeating unit consisting of a single →4)-GlcNAc3NAcA-β(1→ residue. The TUA is terminated with a 4O-acetylated GlcNAc3NAcA residue on the non-reducing end, while the reducing-end residue of GlcNAc3NAcA forms a β(1→4) glycosidic bond with a ribitol-5-phosphate residue. D) Ribitol phosphate units identified in the wild-type and mutant WTA. The WTA contains 1,5-linked ribitol-P residues, a large portion of which is substituted by β-Glc residue in the 2- or 4-position. The mutant WTA has much higher content of a terminal ribitol-5P residue (residue Rc, in bold), presumably equivalent to the terminal Rbo-5P that is substituted by TUA in the wild-type material. E) 1H NMR (top) and DOSY (bottom) spectra of the S. antibioticus wild-type (left) and ΔutrC (right) TUA/WTA preparations. The wild-type DOSY spectrum shows that the TUA and WTA have the same diffusion coefficient, indicating the same size. All WTA signals in the ΔutrC DOSY are also aligned at the same D level, consistent with a size-uniform preparation. Given its lower D, the size of ΔutrC WTA is likely somewhat larger compared to the wild-type [file NIHMS2121434-supplement-11.pdf]

Figure S5

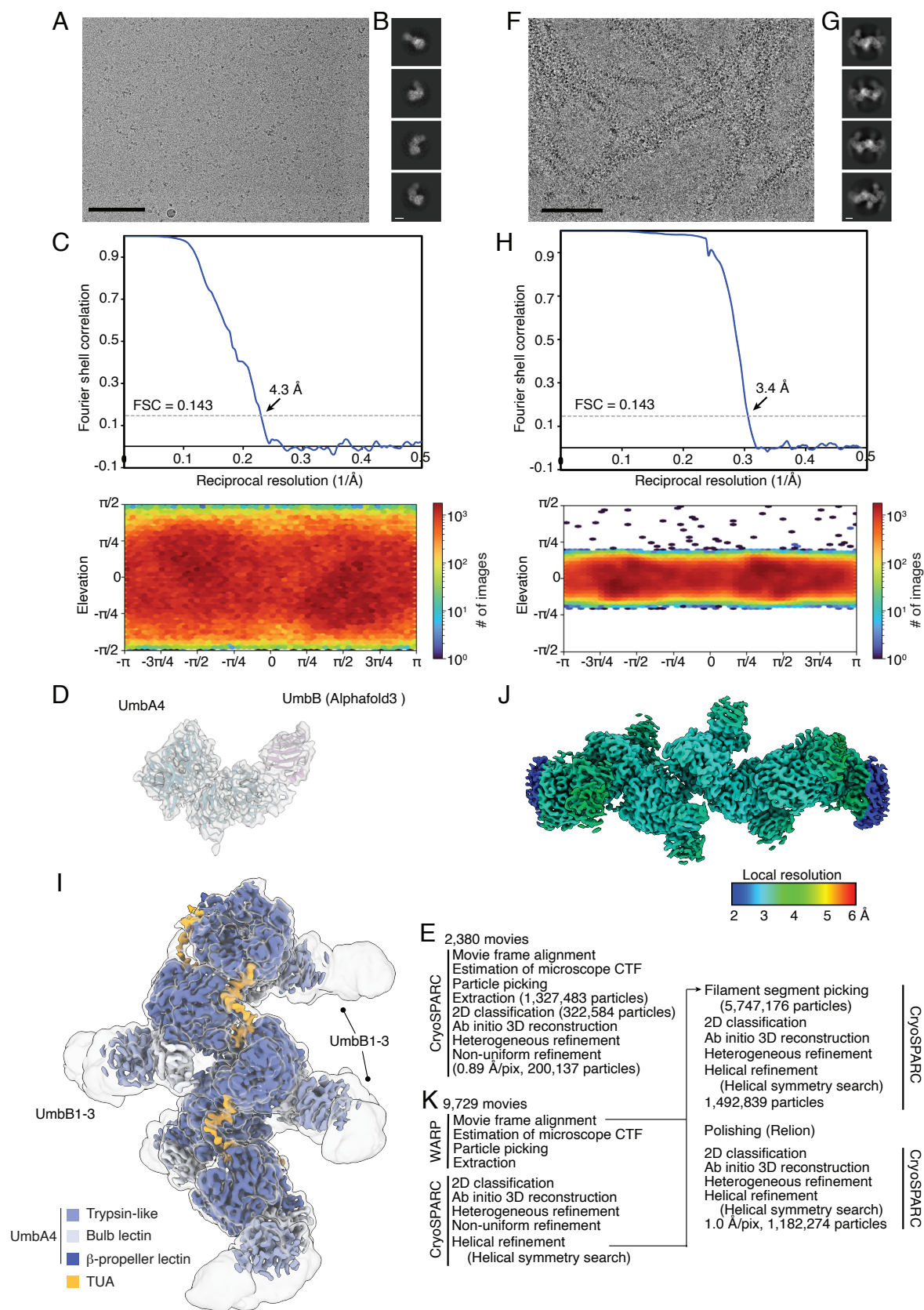

Supplement: 12 — Figure S5. Cryo-EM data processing of the UmbA4 and TUA molecules bound UmbA4 complex datasets, related to Figure 5. A, B, F and G) Representative electron micrographs (A, F) and 2D class averages (B, G) of the UmbA4 complex (A, B) or TUA molecules bound UmbA4 complex (F, G) embedded in vitreous ice. Scale bars: 100 nm (A,F), 20 nm (B,G). C,H) Gold-standard Fourier shell correlation curve of the UmbA4 complex (C) or TUA molecules bound UmbA4 complex (H). The 0.143 cutoff is indicated by a horizontal dashed line. The angular distribution of particle images calculated using cryoSPARC is shown as a heat map below. D) 3D reconstruction of UmbA4 complex that encompassed two proteins (UmbA4 in blue and UmbB in magenta). E,K) Data processing flowchart. CTF: contrast transfer function; NUR: non-uniform refinement. J) Local resolution estimation of TUA molecules bound UmbA4 complex reconstruction calculated using cryoSPARC and plotted on the sharpened maps. I) Unsharpened (transparent) and sharpened (opaque) cryo-EM maps of a helical segment of the TUA–UmbA4–UmbB1–3 complex. Regions encompassing TUA and UmbA4 are colored based on model proximity. [file NIHMS2121434-supplement-12.pdf]

Figure S6

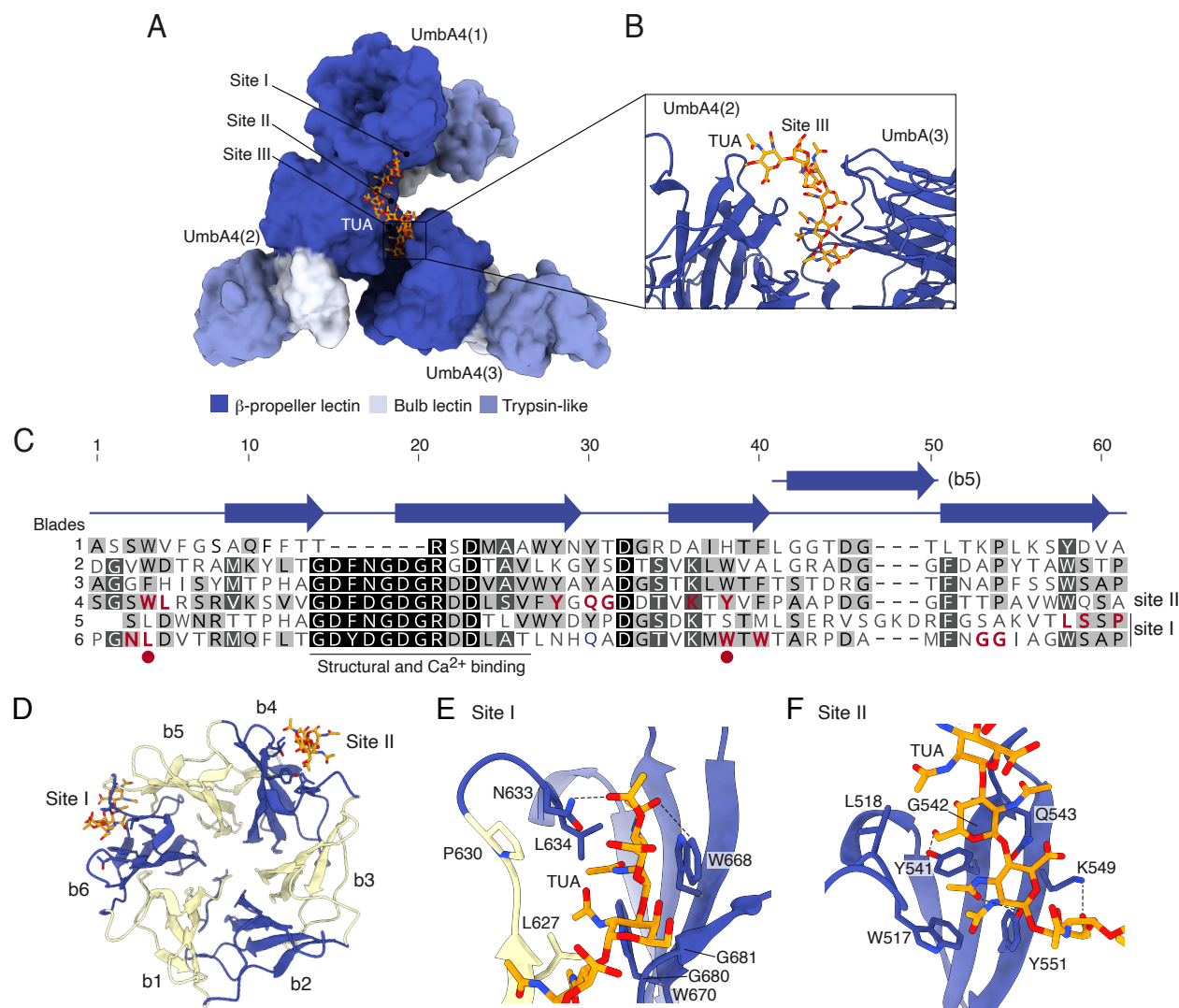

Supplement: 13 — Figure S6. TUA binds at conserved sites on the β-propeller lectin, related to Figure 5. A) Overview of sites I, II, and III on the asymmetric unit containing three UmbA4 protomers. B) Close-up of site III. TUA packs between UmbA4(2) and UmbA4(3) making weak contacts with both protomers. C) Sequence alignment of the β-propeller lectin blades. Secondary structure is indicated above the alignment. Residues that form direct interactions with TUA are indicated (red). Residue positions that interact with TUA in both site I and II are indicated (circle). D) Top-down view of a single UmbA4 molecule indicating sites that make contact with TUA in different protomers in the cryo-EM structure. Individual blades of the β-propeller are labeled (b1–6). E, F) Zoom-in view of UmbA4–TUA interaction sites I (E) and II (F), indicating the contacting amino acids. [file NIHMS2121434-supplement-13.pdf]
