# Supplementary material for: The unique architecture of umbrella toxins permits a two-tiered molecular bet hedging strategy for interbacterial antagonism
Source: Cell. Author manuscript; Available in PMC 2026 Jun 17. (PMC13274773; doi:10.1016/j.cell.2025.10.044)
Supplement: 2 — Document S1. Table S1 and S5. [file NIHMS2121434-supplement-2.pdf]

Table S1. Results from screening sup^{Sc} and sup^{Sg} for growth inhibitory activity against diverse bacteria, related to Figure 1 and Figure 3. Growth was measured at a single timepoint using an ATP quantification-based bacterial cell viability assay. The average ratio (log_2) represents the viability of each strain grown with control supernatant divided by the relevant sup treatment in two biological replicates of the screen. Z-scores were calculated from the average of log_2-transformed ratios from across all strains screened for a given treatment.

Genus and species	Average ratio (log_2)	Z-score	Source
**Screen with sup^{Sc}**			
S. ossamyceticus SAI_001	1.993	0.348	Zhao *et al.*, 2024. PMID: 38632398
S. albogriseolus SAI_072	1.569	0.111	Zhao *et al.*, 2024. PMID: 38632398
S. sp. SAI_056	2.321	0.532	Zhao *et al.*, 2024. PMID: 38632398
S. lannensis SAI_137	0.594	-0.437	Zhao *et al.*, 2024. PMID: 38632398
S. melanosporofaciens	0.906	-0.262	NRRL No. B-12234
S. curacoi ELPA-25	0.857	-0.289	Isolated in this study
S. sp. SAI_169	0.764	-0.341	Zhao *et al.*, 2024. PMID: 38632398
*S. sp.*SAI_098	1.690	0.178	Zhao *et al.*, 2024. PMID: 38632398
*S. sp.*SAI_125	2.747	0.771	Zhao *et al.*, 2024. PMID: 38632398
S. canus SAI_131	2.017	0.362	Zhao *et al.*, 2024. PMID: 38632398
S. venezuelae	-0.375	-0.980	ATCC 10712
S. roseus	-0.202	-0.883	NRRL No. B-3062
S. achromogenes SANT-13	7.845	3.632	Isolated in this study
S. galilaeus BO15-53	1.065	-0.172	Isolated in this study
S. griseoruber YUMA-11	0.692	-0.382	Isolated in this study
S. hypolithicus YUMA-33	0.688	-0.384	Isolated in this study
S. werraensis YUMA-35	0.174	-0.672	Isolated in this study
S. enissocaesilis YUMA-50	0.191	-0.663	Isolated in this study
S. griseolus YUMA-69	0.538	-0.468	Isolated in this study
**Screen with sup^{Sg}**			
S. ossamyceticus SAI_001	-0.409	-0.162	Zhao *et al.*, 2024. PMID: 38632398
S. albogriseolus SAI_072	0.090	0.840	Zhao *et al.*, 2024. PMID: 38632398
S. sp. SAI_056	-0.454	-0.252	Zhao *et al.*, 2024. PMID: 38632398
S. lannensis SAI_137	-0.192	0.273	Zhao *et al.*, 2024. PMID: 38632398
S. melanosporofaciens	-0.662	-0.669	NRRL No. B-12234
S. curacoi ELPA-25	-0.160	0.337	Isolated in this study
S. sp. SAI_169	-0.236	0.186	Zhao *et al.*, 2024. PMID: 38632398
*S. sp.*SAI_098	-0.469	-0.283	Zhao *et al.*, 2024. PMID: 38632398
*S. sp.*SAI_125	-0.468	-0.281	Zhao *et al.*, 2024. PMID: 38632398
S. canus SAI_131	-0.860	-1.067	Zhao *et al.*, 2024. PMID: 38632398
S. ambofaciens SAI_163	-0.884	-1.115	Zhao *et al.*, 2024. PMID: 38632398
S. venezuelae	-0.550	-0.445	ATCC 10712
S. roseus	-0.428	-0.200	NRRL No. B-3062

S. achromogenes SANT-13	-0.488	-0.320	Isolated in this study
S. galilaeus BO15-53	-0.356	-0.056	Isolated in this study
S. griseoruber YUMA-11	-0.139	0.381	Isolated in this study
S. hypolithicus YUMA-33	-0.546	-0.437	Isolated in this study
S. werraensis YUMA-35	-0.792	-0.930	Isolated in this study
S. enissocaesilis YUMA-50	-0.562	-0.469	Isolated in this study
S. griseolus YUMA-69	-0.015	0.629	Isolated in this study
S. ambofaciens SAI_104	0.110	0.880	Zhao *et al*., 2024. PMID: 38632398
S. tendae SAI_182	0.098	0.855	Zhao *et al*., 2024. PMID: 38632398
S. sp. SAI_167	-0.177	0.304	Zhao *et al*., 2024. PMID: 38632398
S. pristinaespiralis	-0.180	0.299	NRRL No. B2958
S. lividans	-0.376	-0.095	NRRL No. B65306
S. ambofaciens	-0.313	0.032	NRRL No. B-2516
S. luteogriseus	-0.556	-0.457	NRRL No. B-12422
S. graminofaciens SAI_110	-0.146	0.367	Zhao *et al*., 2024. PMID: 38632398
S. graminofaciens SAI_175	-0.620	-0.586	Zhao *et al*., 2024. PMID: 38632398
S. sp. SAI_041	-1.359	-2.069	Zhao *et al*., 2024. PMID: 38632398
S. griseorubiginosus SAI_142	-0.445	-0.234	Zhao *et al*., 2024. PMID: 38632398
S. rochei SAI_164	-0.314	0.028	Zhao *et al*., 2024. PMID: 38632398
S. pristinaespiralis SAI_178	-0.575	-0.495	Zhao *et al*., 2024. PMID: 38632398
S. sp. SAI_103	-0.240	0.177	Zhao *et al*., 2024. PMID: 38632398
S. anulatus	-0.211	0.236	NRRL No. B-2000
S. antibioticus	2.049	4.773	NRRL No. B-1701
S. eurocidicus	-0.315	0.027	NRRL No. B-1676

Table S2. Mutations detected in *S. griseus* and *S. antibioticus* clones obtained following ALE in the presence of *S. coelicolor* and *S. griseus* umbrella toxins, respectively, related to Figure 2 and Figure 3.

See excel file

Table S3. Bioinformatic analysis of proteins encoded by the *S. griseus utr* operon, related to Figure 2.

See excel file

Table S4. Chemical shift assignments of carbohydrate and polyol*P* residues in wild-type and Δ*utrC* preparations of TUA–WTA, TUA and WTA from *S. griseus* and *S. antibioticus*, respectively, related to Figure 2 and Figure 3.

See excel file

Table S5. Cryo-EM data collection, refinement and validation statistics, related to Figure 5.

	UmbA4	TUA bound UmbA4<br>(EMDB-70396)<br>(PDB 9OEE)
**Data collection and processing**		
Magnification	45,000x	105,000x
Voltage (kV)	200	300
Electron exposure (e–/Å^2)	47	40
Defocus range (μm)	-0.2 - -3.0	-0.2 - -3.0
Pixel size (Å)	0.89	0.835
Symmetry imposed	C1	Helical (Twist: 208.86° Rise: 33.13 Å)
Initial particle images (no.)	1,327,483	5,747,176
Final particle images (no.)	200,137	1,182,274
Map resolution (Å)	4.3	3.3
FSC threshold	0.143	0.143
**Refinement**		
Initial model used (PDB code)		Alphafold-generated
Model resolution (Å)		3.6
FSC threshold		0.5
Map sharpening *B* factor (Å^2)		-147
Model composition		
Non-hydrogen atoms		26010
Protein residues		3365
Ligands		60
B factors (Å^2)		
Protein		54.04
Ligand		19.96
R.m.s. deviations		
Bond lengths (Å)		0.002
Bond angles (°)		0.571
Validation		
MolProbity score		1.34
Clashscore		4.59
Poor rotamers (%)		0.79
Ramachandran plot		
Favored (%)		97.47
Allowed (%)		2.38
Disallowed (%)		0.15

Table S6. Diversity of UmbA lectin domains and UmbC toxin domains within *Streptomycetaceae* family, related to Figure 6.

See excel file

Table S7. Primers used in this study, related to Key Resources Table.

See excel file
